# Inhibition of CPT1a as a prognostic marker can synergistically enhance the antileukemic activity of ABT199

**DOI:** 10.1186/s12967-021-02848-9

**Published:** 2021-04-29

**Authors:** Shihui Mao, Qing Ling, Jiajia Pan, Fenglin Li, Shujuan Huang, Wenle Ye, Wenwen Wei, Xiangjie Lin, Yu Qian, Yungui Wang, Xin Huang, Jiansong Huang, Jinghan Wang, Jie Jin

**Affiliations:** 1 Department of Hematology, The First Affiliated Hospital, Zhejiang University College of Medicine, No. 79 Qingchun Road, Hangzhou, Zhejiang People’s Republic of China; 2 Institute of Hematology, Zhejiang University, Hangzhou, People’s Republic of China; 3 Key Laboratory of Hematologic Malignancies, Diagnosis and Treatment, Hangzhou, Zhejiang People’s Republic of China; 4 Department of Hematology, The First Affiliated Hospital of University of Science and Technology of China, Hefei, People’s Republic of China

**Keywords:** ABT199, Carnitine palmitoyltransferase 1, Fatty acid oxidation, Oncogene, Acute myeloid leukemia

## Abstract

**Background:**

Fatty acid oxidation (FAO) provides an important source of energy to promote the growth of leukemia cells. Carnitine palmitoyltransferase 1a(CPT1a), a rate-limiting enzyme of the essential step of FAO, can facilitate cancer metabolic adaptation. Previous reports demonstrated that CPT1a acts as a potential molecular target in solid tumors and hematologic disease. However, no systematic study was conducted to explore the prognostic value of CPT1a expression and possible treatment strategies with CPT1a inhibitor on acute myeloid leukemia (AML).

**Methods:**

The expression of CPT1a in 325 cytogenetically normal AML (CN-AML) patients was evaluated using RT-PCR. The combination effects of ST1326 and ABT199 were studied in AML cells and primary patients. MTS was used to measure the cell proliferation rate. Annexin V/propidium iodide staining and flow cytometry analysis was used to measure the apoptosis rate. Western blot was used to measure the expression of Mcl-1. RNAseq and GC-TOFMS were used for genomic and metabolic analysis.

**Results:**

In this study, we found AML patients with high CPT1a expression (n = 245) had a relatively short overall survival (P = 0.01) compared to patients in low expression group (n = 80). In parallel, downregulation of CPT1a inhibits proliferation of AML cells. We also conducted genomic and metabolic interactive analysis in AML patients, and found several essential genes and pathways related to aberrant expression of CPT1a. Moreover, we found downregulation of CPT1a sentitized BCL-2 inhibitor ABT199 and CPT1a-selective inhibitor ST1326 combined with ABT199 had a strong synergistic effect to induce apoptosis in AML cells and primary patient blasts for the first time. The underlying synergistic mechanism might be that ST1326 inhibits pGSK3β and pERK expression, leading to downregulation of Mcl-1.

**Conclusion:**

Our study indicates that overexpression of CPT1a predicts poor clinical outcome in AML. CPT1a-selective inhibitor ST1326 combined with Bcl-2 inhibitor ABT199 showed strong synergistic inhibitory effects on AML.

**Supplementary Information:**

The online version contains supplementary material available at 10.1186/s12967-021-02848-9.

## Background

Acute myeloid leukemia (AML) is a group of the heterogeneous disease characterized by the clonal proliferation of immature myeloid cells [1]. For young patients, a combination of cytarabine and anthracycline has been used as the standard induction regimen over the last four decades [2, 3]. While, recently some novel drugs like Bcl-2 inhibitor are proved to be effective for elder AML patients. Although the advances in the new drugs for AML treatment are impressive, the 5-year overall survival rate is frustratingly low for adults (25%) and elderly patients (10%) [4]. Thus, research on novel drugs and rational combination therapies is imperative.

Fatty acid oxidation (FAO) is an important source of NADH, FADH2, NADPH and ATP fueling tumor growth in conditions of metabolic stress [5]. Carnitine palmitoyltransferase 1(CPT1) is a protein that catalyzes the rate-limiting step of FAO, controlling FAO directly [6]. Among CPTI family, CPT1a is the most prevailing enzyme because of its wider distribution and better sensibility to their inhibitor malonyl-CoA [7]. Targeting CPT1a has shown remarkable anti-leukemia activity: A novel CPT1a inhibitor ST1326 has been proved effective on leukemia cell lines and primary cells obtained from patients with hematologic malignancies [8]. However, there is not yet a study to exclusively evaluate the prognostic value of CPT1a expression and possible combinational strategy with CPT1a inhibitor on AML.A majority of leukemia cells have a survival advantage over normal cells because they fail to undergo apoptosis [9]. Anti-apoptotic Bcl-2 protein is one such protein that is overexpressed in many cancers, which makes it an ideal target for cancer therapy [9]. Venetoclax (ABT199) is an oral and highly selective bioavailable inhibitor targeting the BH3 domain of Bcl-2 specifically [10]. Compared to other drugs in its class, venetoclax has lower hematological toxicity [11]. To date, it has been approved for the treatment of first-line and relapsed/refractory chronic lymphocytic leukemia (CLL) and AML [12]. Unfortunately, despite its promising results in hematologic malignancies, intrinsic resistance is still a big problem [13]. Myeloid cell leukemia sequence 1 (Mcl-1) is an antiapoptotic protein that plays a key role in promoting cell survival in AML. Overexpression of Mcl-1 is associated with treatment resistance and poor prognosis [14]. Previous study show that ABT199 (Venetoclax) has promising antileukaemic activity in AML therapy but increasing Mcl-1 limits its effect [15].

In this study, we found higher CPT1a levels were associated with poor prognosis and downregulation of it inhibited proliferation of AML cells, providing direct evidence for CPT1a as a prognostic biomarker for AML. The genomic and metabolic patterns identified several critical pathways to decipher its role of adverse prognostic biomarker. Moreover, our group combined ST1326 with ABT199 in AML cell lines and primary AML cells to identify the synergistic effect through downregulation of Mcl-1. We found ST1326 inhibited pGSK3β and pERK to prevent up-regulation of Mcl-1 induced by ABT199 effectively. Here, we provide a proper combinational therapy strategy to remedy the limited effect of CPT1a selective inhibitor, in the meanwhile, also solve the problem of resistence of ABT199.

## Materials and methods

### Drugs

MG132 were purchased from Selleck Chemicals (Houston, Texas, USA). ST1326 and ABT199 was purchased from Sigma-Aldrich (St Louis, MO, USA).

### Cell culture

HL-60 (acute promyelocytic leukemia cell line), THP-1 (acute monocytic leukemia cell line), OCI-AML2 (acute myelomonocytic leukemia cell line) and OCI-AML3 (acute myelomonocytic leukemia cell line) cell lines were purchased from Shanghai Cell Bank of the Chinese Academy of Sciences. KASUMI-1 (acute myeloblastic leukemia cell line) cell line was gifted by Professor Chen Saijuan (Shanghai Institute of Hematology, Shanghai, China). These cells were cultured in RPMI 1640 medium supplemented with 10% fetal bovine serum (FBS) (Gibco) at 37 °C in a humidified incubator containing 5% CO2. MV4-11(acute myelomonocytic leukemia cell line) and MOLM-13(acute monocytic leukemia cell line) cell lines were a kind gift from Professor Ravi Bhatia (City of Hope National Medical Center, Duarte, CA, USA). These two cell lines were cultured in Iscove's Modified Dulbecco's Medium (IMDM) supplemented with 10% (FBS). Diagnostic AML patient samples were purified by standard Ficoll‐Hypaque (Sigma-Aldrich) density centrifugation, then cultured in RPMI 1640 with 10% FBS.

### Clinical samples

Clinical data were collected from Zhejiang Institute of Hematology, China. Informed consent was provided from all patients according to institutional guidelines. This study was approved by the Ethics Committee of the First Affiliated Hospital of Zhejiang University. From July 2010 to July 2016, 325 patients and 8 healthy controls were included in this study. Healthy controls are age matched individuals without any evidence of hematological disease. Cytogenetically normal acute myeloid leukemia (CN-AML) was defined as AML with the karyotype 46 XY [20] or 46 XX [20] in all 20 metaphase cells analyzed. Gene mutations were analyzed by whole gene sequencing. Patient characteristics were summarized using descriptive statistics, which include frequency, counts, median, and range.

### Cell viability assay

AML Cells were seeded in 96-well plates at 1–2 × 10^4^ (AML cell lines) or 1 × 10^5^ (primary AML cells) per well. Then cells were treated with variable concentrations of ST1326 and/or ABT199 for 48 h. Next, 10 μl MTS solution (Promega, Madison, WI) was added to each well. Cells were incubated for 4 h at 37 °C in a humidified incubator containing 5% CO_2_. Finally, cells at plates were assessed at a wavelength of 490 nm. The dose–effect curves and combination index values were determined by CalcuSyn analyses. For the AML cell lines, experiments were performed 3 independent times in triplicate, while primary patient sample experiments were performed once in triplicate due to limited sample.

### RNA knockdown in human leukemia cell lines

To knock down CPT1a in human leukemia cell lines, short hairpin RNAs (shRNAs) were designed and cloned into a modified psi-LVRU6GP-shRNA plasmid. The sequences of sh1 was GCTCTTAGACAAATCTATCTC. The sequence of sh2 was GCCTTTGGTAAAGGAATCATC. The sequences of NC shRNA were ACAGAAGCGATTGTTGATC. These vectors were then packaged in human embryonic kidney 293T cells. The supernatant containing packaged viral particles was collected at 48 h and 72 h. AML cell lines were transfected with the shRNA or control lentiviruses and incubated for 72 h. Next, cells were continuously cultured in the medium containing 1.0 μg/mL puromycin.

### Growth curve assay

Cells were seeded in 96-well plates (1.0 × 10^4^ cells per well), blank medium as a control, 10 μL of MTS solution (Promega CellTitre96) (5 mg/mL) were added to each well at 0 h, 24 h, 48 h and 72 h, and the cells were incubated for an additional 4 h at 37℃, the absorbance was measured at 490 nm.

Annexin V/propidium iodide staining and flow cytometry analysis.

AML cells were treated with ST1326 or ABT199, alone or in combination for 48 h. Then cells were co-stained with Annexin VFITC and Propidium Iodide (PI) for 15 min using an apoptosis detection kit (Beckman Coulter, Brea, CA, USA) in the dark. Apoptotic cells were analyzed by flow cytometry using FACScan™ flow cytometer (Becton Dickinson, San Diego, CA, USA). Results are expressed as percent annexin V + cells.

### Western blot analysis

Cells were lysed in radioimmunoprecipitation (RIPA) buffer (Cell Signaling Technology) on ice for 30 min. After centrifugation of the cell lysate at 12,000×*g* for 15 min at 4 °C, Protein concentration of the cellular supernatant was determined using BCA reagent (BBI life science, Shanghai, China). Cell lysates were then loaded onto 10% SDS-PAGE (Life Technologies, Carlsbad, CA, USA). After electrophoresis, proteins were transferred to PVDF membrane (Millipore, Billerica, MA, USA). Then, the membranes were blocked with 5% non-fat milk for 1 h and incubated with primary antibodies overnight at 4 °C. Membranes were incubated with secondary antibodies (Cell Signaling Technology) for 1 h at room after washing three times with TBST buffer temperature. The target proteins were visualized using an ECL detection kit (Amersham, Little Chalfont, UK) and analyzed using Image Lab™ software (Bio-Rad Laboratories, Hercules, CA, USA).

Primary antibodies for immunoblotting were purchased from the following sources: caspase3, cleaved caspase3, PARP, Bad, Bax, BCL-2, BCL-xl, BIM, p-ERK, ERK, p-Mcl1Thr163, p-GSK3β, GSK3β, AKT, p-AKT, GAPDH, β-tubulin and β-actin antibodies were purchased from Cell Signaling Technology (Beverly, MA, USA).

### Real-time RT-PCR

Total RNA was isolated from the AML cells using TRIzol reagent according to the manufacturer's instructions. Reverse transcription was performed using the RNAPCR core kit (Life Technologies, Paisley, UK). Real-time quantitative PCR was performed on iQ5 System (Bio-Rad, Hercules, CA) using a SYBR Green qPCR master mix with GAPDH as an internal control. Primer sequences used are listed in following:

Mcl-1,5′-AAGAGGCTGGGATGGGTTTGTG-3′(forward),5′-TTGGTGGTGGTGGTGGTTGG-3′(reverse); CPT1a,5′-ATCAATCGGACTCTGGAAACGG-3′(forward), 5′-TCAGGGAGTAGCGCATGGT-3′(reverse); GAPDH,5′-GGAGCGAGATCCCTCCAAAAT-3′(forward),5′-GGCTGTTGTCATACTTCTCATGG-3′(reverse).

### Sample preparation and GC-TOFMS analysis

12 bone marrow samples with aberrant CPT1a expression collected at disease diagnosis were stored frozen at − 80 °C until use. 10^7^ cells of each bone marrow sample were added into a 1.5 mL of tube followed by the addition of 400 μL of acetone for protein precipitation. The mixture was stirred by vortex for 30 s and centrifuged at 10,000 rpm for 10 min. A 400-μL supernatant was transferred to a 500 μL of glass tube and dried under vacuum. The dried analytes were dissolved in 80 μL of methoxylamine hydrochloride (15 mg/mL, dissolved in pyridine) for 90 min at 30 °C and then silylated with 80 μL N,O-bis-trimethylsilyl-trifluoroacetamideand Trimethylchlorosilane (in a ratio of 99:1) (Supelco) for 2 h at 70 °C. Each 70-μL aliquot of hexane was added to the derivatization bottles. After the sample was stirred for 1 min and kept at room temperature for an hour, 1-μL aliquot of the solution was injected into a PerkinElmer gas chromatography coupled with a TurboMass-Autosystem XL mass spectrometer (PerkinElmer, Inc.) in the splitless mode. A DB-5MS capillary column coated with 5% Diphenyl cross-linked 95% dimethylpolysiloxane (30 m × 250 μm i.d., 0.25-μm film thickness; Agilent J&W Scientific, Folsom, CA) was used for separation. Both the injection temperature and the interface temperature were set to 260 °C, and the ion source temperature was adjusted to 200 °C. Initial GC oven temperature was set at 80 °C for 2 min after injection, and was raised up to 285 °C with 5 °C/min and maintained at 285 °C for 7 min. Helium at a flow rate of 1 mL/min was used as the carrier gas. The measurements were made with electron impact ionization (70 eV) in the full scan mode (m/z 30–550). A total of 71 metabolites were identified by the comparison with the internal library built with the standard reference compounds.

### Gene expression arrays

22 BM samples of CN-AML patients were used to assess the mRNA expression profiling. Briefly, rRNAs was removed from total RNA using Ribo-Zero rRNA Removal Kits (Illumina, USA) following the manufacturer's instructions. RNA quality was evaluated using a Nanodrop ND-1000 (Thermo Fisher Scientific, Waltham, MA, USA). Transcriptome high throughput sequencing was done by Cloud-Seq Biotech (Shanghai, China). Briefly, total RNA was used for removing the rRNAs using Ribo-Zero rRNA Removal Kits (Illumina, USA) following the manufacturer's instructions. RNA libraries were constructed by using rRNA-depleted RNAs with TruSeq Stranded Total RNA Library Prep Kit (Illumina, USA) according to the manufacturer’s instructions. Libraries were controlled for quality and quantified using the BioAnalyzer 2100 system (Agilent Technologies, USA). Paired-end reads were harvested from Illumina HiSeq 4000 sequencer, and were quality controlled by Q30. After 3′ adaptor-trimming and low quality reads removing by "cutadapt" software (v1.9.3). The high quality trimmed reads were used to mRNA analyses. The high quality reads were aligned to the human reference genome (UCSC hg19) with hisat2 software. Then, guided by the Ensembl gtf gene annotation file, cuffdiff software (part of cufflinks) was used to get the FPKM as the expression profiles of mRNA.

### Statistical analysis

AML patient characteristics were summarized using descriptive statistics, which included frequency counts, median and interquartile range. Categorical variables were compared using Fisher’s exact test, and continuous variables were analyzed using a nonparameter T-test. OS was defined as time from the date of diagnosis until death due to any cause or the last follow-up. Univariate and multivariate analyses with a Cox proportional hazards models were performed to assess significant predictors. The proportional-hazards assumption was checked for each variable before fitting Cox models. We searched for candidate mRNAs related to aberrant CPT1a expression using the "edgeR". The differently expressed metabolites were identified by the " multtest ". Integrative analysis of metabolite and mRNA was performed in silico using the online platform(https://www.metaboanalyst.ca/MetaboAnalyst/home.xhtml). Data were analyzed using GraphPad Prism 8.0 software. CalcuSyn software (Biosoft, Cambridge, UK) was used to calculate the combination index (CI). All other statistical analyses were conducted using R software, version 3.6.1 (www.r-project.org). The two-sided level of significance was set at p-value < 0.05.

## Results

### Overexpression of CPT1a predicts poor clinical outcome in Chinese AML patients and downregulation of it inhibits proliferation of AML cells

**Fig.1:**
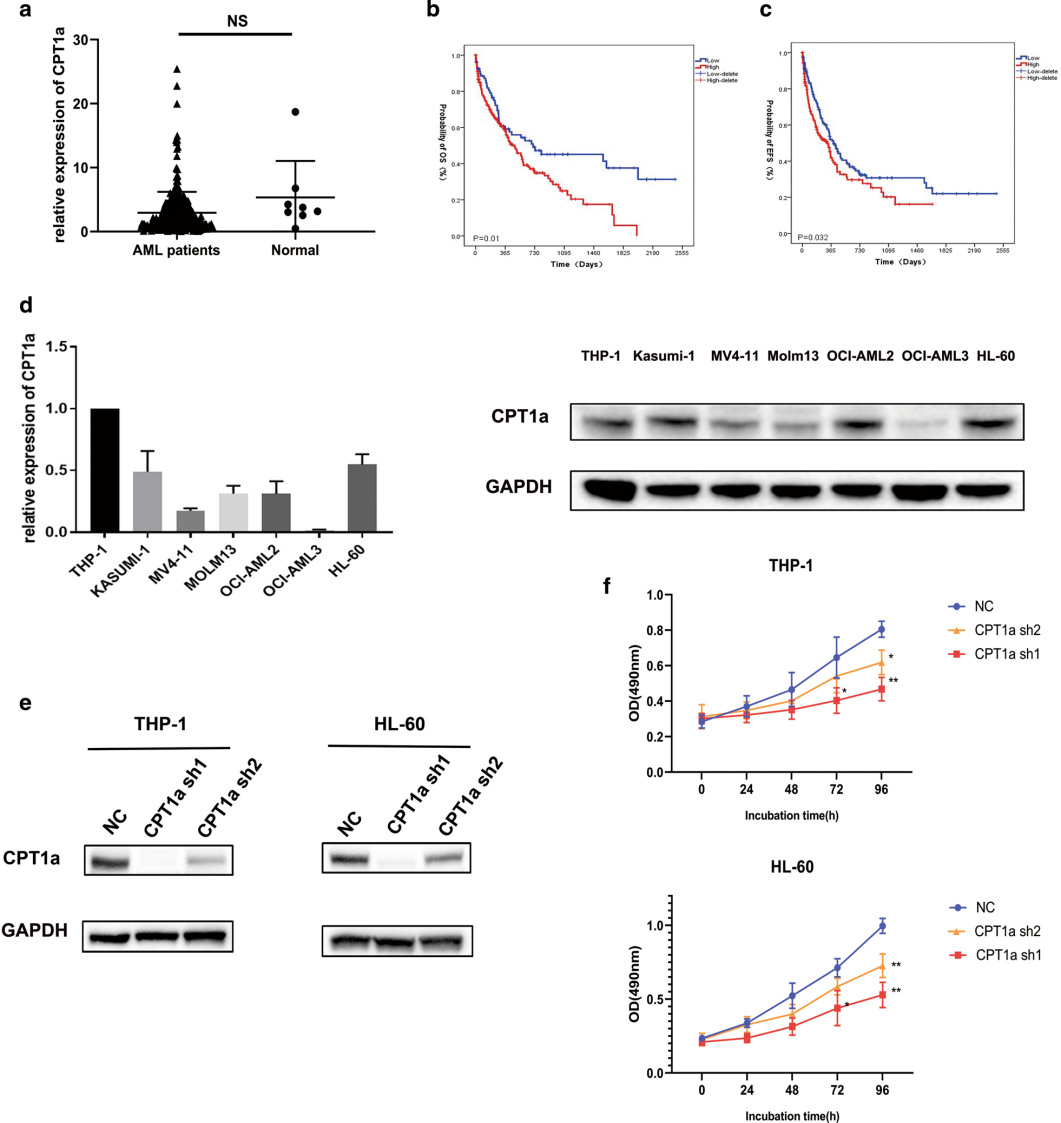
**a** qRT-PCR analysis of CPT1a mRNA expression (mean ± SEM)in normal cells (n = 8) and CN-AML samples (n = 325) (t-test). **b** Kaplan–Meier analysis of overall survival (OS) according to CPT1a mRNA expression in primary blasts from 325 AML patients. **c** Kaplan–Meier analysis of event free survival (EFS) according to CPT1a mRNA expression in primary blasts from 325 AML patients. **d **qPCR and Western blotting analysis of CPT1a expression in human AML cell lines. **e** Western blotting analysis of the CPT1a protein level in THP-1 and HL-60 cells transduced with 2 different CPT1a shRNAs. The NC shRNA was used as a knockdown control. **f** Cell viability at 0 h, 24 h, 48 h and 72 h in THP-1 and HL-60 cells transduced with 2 different CPT1a shRNAs

First, we measured CPT1a mRNA expression of 325 AML patients and 8 normal people by real-time PCR. CPT1a mRNA expression was similar between primary AML samples (n = 325) and normal BM cells from healthy donors (n = 8) (Fig. 1a). According to the mRNA expression level of CPT1a, patients were classified into high expression group (n = 245, 75%) and low expression group (n = 80, 25%). We found that there was no statistical correlation between CPT1a expression and variables of gender, age, BM blasts, WBC levels, hemoglobin levels, platelet counts, FAB classifications and genes mutations (Table 1). In survival analyses, high CPT1a expression (n = 245) had a relatively short overall survival (OS) (P = 0.01, log-rank test) and event free survival (EFS) (P = 0.08, log-rank test) compared to patients in low expression group (n = 80) (Fig. 1b and c). And in the multivariable analysis, high CPT1a expression is associated with poor overall survival (OS) [HR (95% CI), 1.674 (1.097, 2.557); P = 0.017, Table 2] after adjusting age, WBC, ENL classification, DMNT3a and IDH1, IDH2 mutations. The probability of event-free survival (EFS) was not significantly different in the multivariable analysis [HR (95% CI), 1.412 (0.965, 2.066); P = 0.076 Additional file 1: Table S1]. Next, RT-qPCR and Western blotting were performed to detect the mRNA and protein expression levels of CPT1a in a panel of human AML cell lines (Additional file 1: Table S2). The results showed that high expression levels of CPT1a were detected in THP-1, HL-60, KASUMI-1 and AML-OCI2 cell lines and that the lowest expression level was detected in OCI-AML3 cell line (Fig. 1d).We silenced the expression of CPT1a by introducing lentivirus-encoded shRNAs into THP-1 and HL-60 cell lines. ShRNAs can efficiently decrease the expression of CPT1a (Fig. 1e) and cause a significant reduction in proliferation of both THP-1 and HL-60 cell lines (Fig. 1f).

**Table 1:** Characteristics of CN-AML patients by high and low CPT1a expression

	CPT1a expression		P-value
	LOW expression (N = 80)	High expression (N = 245)	
Number (%)	80 (25%)	245 (75%)	
Male, n (%)	53 (67.1)	137 (56.6)	0.13
Age, median (IQR),years	49.00 (36.00, 61.00)	56.00 (39.75, 65.00)	0.054
BM blast, median (IQR),%	60.00 (29.00, 81.00)	68.00 (43.00, 81.00)	0.179
WBC, median (IQR), ×10^9^/L	14.70 (3.65, 64.68)	10.50 (2.38, 45.98)	0.336
HB, median (IQR), g/L	89.90 (67.75, 105.75)	84.00 (67.75, 102.25)	0.360
PLT, median (IQR),×10^9^/L	45.00 (22.75, 78.00)	49.00 (26.00, 94.75)	0.199
FAB classification, n (%)			0.996
M0	8 (10.0)	22 (9.0)	
M1	7 (8.8)	19 (7.8)	
M2	39 (48.8)	122 (49.8)	
M3	1 (1.2)	3 (1.2)	
M4	3 (3.8)	10 (4.1)	
M5	20 (25.0)	63 (25.7)	
M6	2 (2.5)	4 (1.6)	
Genes mutations, n (%)			
FLT3ITD	11 (14.1)	49 (20.9)	0.190
CEBPA^DM1^	10 (13.9)	30 (13.8)	1
NPM1	19 (25.0)	63 (28.0)	0.657
DNMT3A	5 (7.1)	33 (15.8)	0.073
IDH1	19 (26.0)	42 (20.3)	0.325
IDH2	7 (10.8)	33 (16.3)	0.323
ELN favorable group, n (%)			
Treatment, n (%)^2^			
CPT1A, median (IQR)	0.58 (0.30, 0.78)	2.79 (1.69, 4.66)	< 0.001

WBC: white blood cell; HB: hemoglobin; PLT: platelet counts; BM: bone marrow; FAB: French–American–British classification systems

^1^DM: Double−allele

^2^The protocols used for induction therapy in different groups including HAA, homoharringtonine−based treatment (homoharringtonine 2 mg/m2/day for 3 days, cytarabine 75 mg/m^2^ twice daily for 7 days, aclarubicin 12 mg/m^2^ daily for 7 days) regimen; DA, daunorubicin 45 mg/m^2^ daily for 3 days and cytarabine 100 mg/m^2^ daily for 7 days; IA, idarubicin 6–8 mg/m^2^ daily for 7 days and aclarubicin 20 mg/m^2^ daily for 5 days. IQR, interquartile. BMT: bone marrow transplantation. ELN (European leukemia Net) favorable genotype represents NPM1 mutant and FLT3−ITD negative or double allele CEBPA mutations

**Table 2:** Univariate and multivariate overall survival analyses in CN-AML

	Univariate analysis		Multivariate analysis	
Variables	P-value	HR (95% CI)	P-value	HR (95% CI)
CPT1A expression	0.005	1.693 (1.167,2.454)	0.017	1.674 (1.097,2.557)
age	< 0.001	1.034 (1.024,1.044)	< 0.001	1.04 (1.028,1.052)
WBC	< 0.001	1.004 (1.002,1.006)	< 0.001	1.006 (1.003,1.008)
ENL favorable group	< 0.001	0.499 (0.339,0.734)	< 0.001	0.319 (0.202,0.505)
DNMT3a	0.002	1.971 (1.282,3.03)	0.204	1.341 (0.853,2.11)
IDH1	0.084	1.386 (0.957,2.007)	0.037	1.626 (1.03,2.567)
IDH2	0.736	1.081 (0.686,1.703)	0.73	0.92 0.574,1.476)

HR: hazard ratio; CI: confidence interval

### Associations of metabolic and genomic expression profiles with aberrant CPT1a expression

**Fig. 2:**
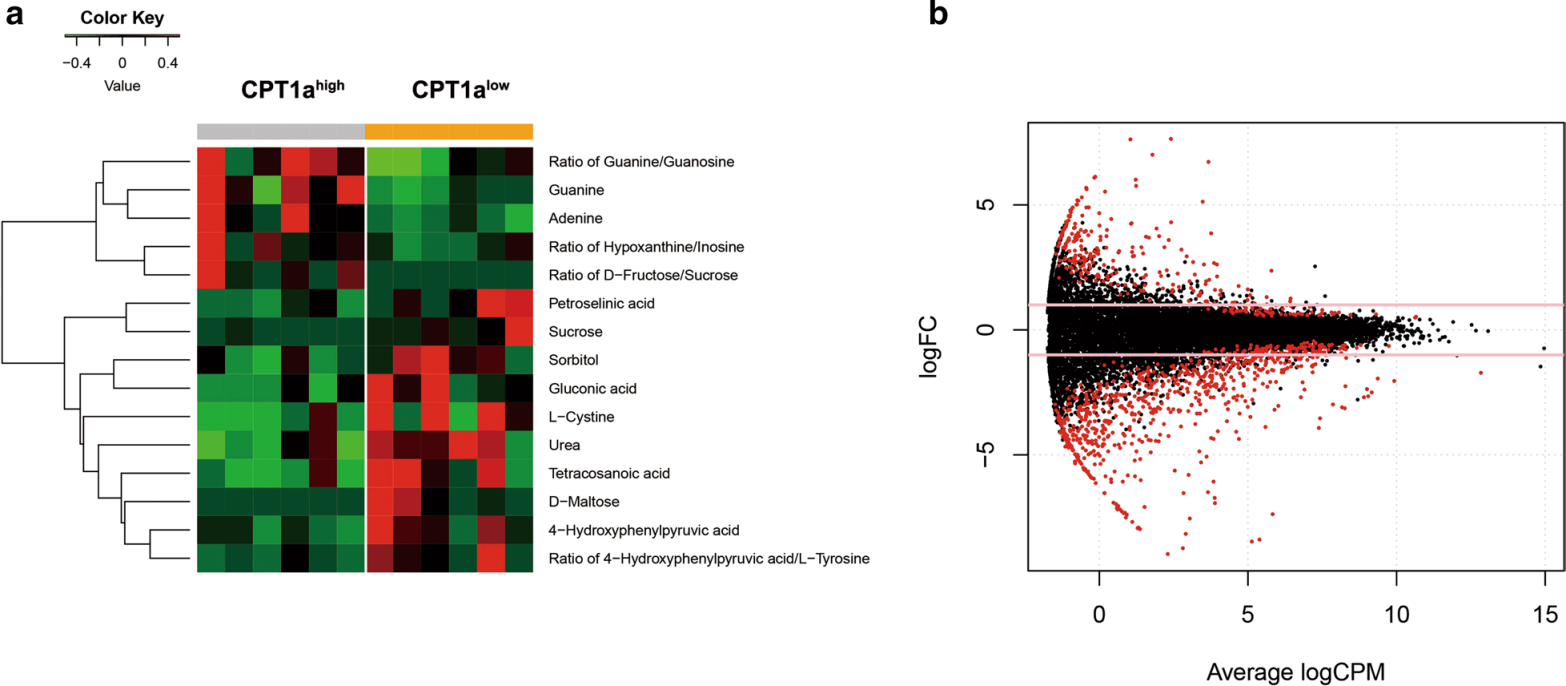
**a** Global metabolomics profile between high and low expression of CPT1a of 12 patients. **b** Volcano plot of differential gene profiles between high and low expression of CPT1a of 22 patients

Frozen-fresh clinical samples (Additional file 1: Table S3) balanced on the clinical and molecular factors were used to explore the biological insight by metabolomics and mRNA expression profiling. As expected, we observed a decrease of fatty acid like tetracosanoic acid in CPT1a high expression group comparing to low expression group, which confirms that CPT1a can facilitate FAO to provide energy fueling tumor growth (Fig. 2a). Additionally, amino acid levels and urea cycle intermediates were decreased accompanied by increases in nucleotide synthesis, suggesting the utilization of more glucose and amino acids for the purpose of nucleotide synthesis and cellular proliferation (Fig. 2a). 233 up-regulated and 673 down-regulated genes were identified as to be significantly associated with CPT1a expression (P < 0.05 and |logFC|> 1) (Additional file 1: Table S4). These aberrant genes were shown in Fig. 2b. The up-regulated genes included: (1) genes involving in tumorigenesis promoters (such as RASGRP3, ErbB3, HOXC8; (2) genes correlating with energy metabolism (such as FFAR3,ND3); (3) Leucocyte Receptor Complex:LAIR2,LILRB1,LILRA4.The down-regulated genes included: (1) immune system activators such as ICAM1,CD40; (2) Hematopoietic tumor suppressor such as KLF5, ALOX5. (3) Bcl-2 family (Bcl-2A1, Bcl-XL, Bcl-2L15). Then we conducted the metabolic and genomic integration analysis. As a result, 52 pathways were found to be significantly associated with aberrant expression of CPT1a (P < 0.05), including: (1) Leukemia-related pathways, such as Human T-cell leukemia virus 1 infection, Signaling pathways regulating pluripotency of stem cells; (2) Apoptosis; (3) signal transduction pathway, such as cGMP-PKG signaling pathway, cAMP signaling pathway; (4) Other molecule-related pathways, such as TGF-beta signaling pathway, NF-kappa B signaling pathway, MAPK signaling pathway, PI3K-Akt signaling pathway. (5) pathways in other cancers, such as melanoma, small cell lung cancer, prostate cancer and bladder cancer (Additional file 1: Table S5).

### Inhibition of CPT1a can synergistically enhance the antileukemic activity of ABT199

**Fig. 3:**
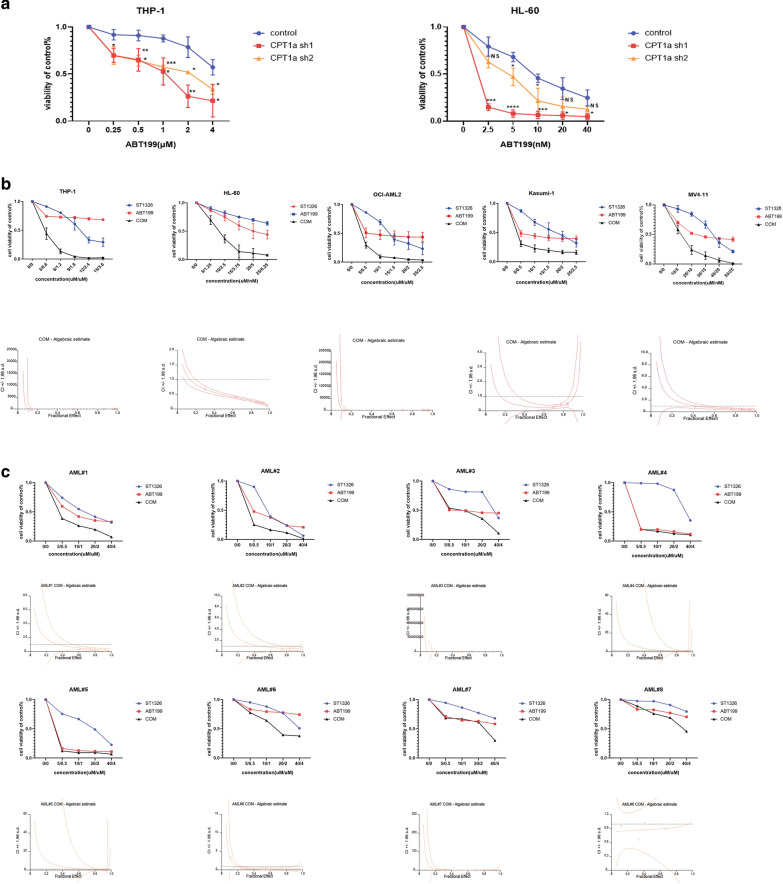
Cell viability after treatment with ABT199 in THP-1 and HL-60 cells transduced with 2 different CPT1a shRNAs **a** **b,c**Cell viability after treatment with ST1326, ABT199 or combination in AML cell lines (**b**, up panel) and primary AML cells (**c**, up panel) measured by MTS assay. The combination index (CI) ( **b,c** down panel) was calculated using CalcuSyn software. The data are presented as mean ± SD format least three independent experiments for cell lines. For patient sample, the MTS assay was performed once due to limited sample

Aberrant expression of CPT1a is related to pro-survival Bcl-2 family protein and apoptosis pathway as mentioned above. Previous study also demonstrates that CPT1 can interact with Bcl-2 [16] and the truncated form of the proapoptotic Bcl-2 family member Bid (tBid) decreases CPT1 activity. And overexpression of Bcl-2 antagonizes this effect through direct interaction with CPT1a [17]. So we hypothesized that inhibiton of CPT1a have a combinational effect with ABT199. We silenced the expression of CPT1a and found the knockdown of CPT1a made THP-1 and HL-60 cell lines more sensitive to ABT199 (Fig. 3a). A novel CPT1a inhibitor ST1326 has been proved effective on leukemia cell lines and primary cells obtained from patients with hematologic malignancies [8]. In order to better connect to clinical application, THP-1, HL-60, Kasumi-1, MV4-11 and OCI-AML2 cell lines were used to evaluate the combinatorial effect of ST1326 and ABT199. The results showed that exposure to both ST1326 and ABT199 single treatment could inhibit AML cell proliferation in a dose-dependent manner. What’s more, co-administration of ST1326 and ABT199 resulted in a further increased inhibition of cell proliferation in THP-1, HL-60, Kasumi-1, MV4-11 and OCI-AML2 cell lines (Fig. 3b). Synergies (CI < 1.0) were also observed in both ABT199-sensitive primary cells and ABT199-resisitent primary cells(Fig. 3c). The characteristics of the patient samples were presented in Table 3. The dose–effect curves were determined by CalcuSyn analyses (Fig. 3b and c, down panel). The CI values were presented in Table 4. We demonstrated that ST1326 combined with ABT199 had a strong synergistic effect (CI < 1.0) in AML cell lines and primary AML cells in vitro.

**Table 3:** Characteristics of primary AML patients

	Diagnose	Gender	Age (year)	FAB type	Cytogenetics	Molecular
AML#1	Refractory	Female	30	M2a	46, XX	NARAS
AML#2	De novo	Female	40	NA	46, XX	BCR/ABL BCORL1 KMT2C
AML#3	Refractory	Male	63	M2	46, XY	RUNX1
AML#4	De novo	Female	64	M2	46, XX	SH2B3
AML#5	Refractory	Female	67	M4	46, XX	
AML#6	Refractory	Female	33	M5	46, XX	FLT3-ITD NPM1
AML#7	De novo	Female	33	M4	46, XX	WT1 MLL-AF9
AML#8	Refractory	Female	73	M0	46, XX	CBFB-MYH11 WT1

**Table 4:** Combination Index Values of AML cell lines and AML patients

AML cell lines	Combination index values		
	ED50	ED75	ED90
THP-1	0.26890	0.24955	0.23277
Kasumi-1	0.25727	0.32034	0.87657
MV4-11	0.84190	0.51618	0.39560
OCI-AML2	0.63777	0.23040	0.27239
HL-60	0.48821	0.32436	0.21859

Patient	Combination index values		
	ED50	ED75	ED90
AML#1	0.67927	0.36933	0.23918
AML#2	0.94233	0.60820	0.55849
AML#3	1.39321	0.18723	0.18043
AML#4	2.26997	0.68618	0.29174
AML#5	0.18060	0.02656	0.00498
AML#6	0.88419	0.91904	0.96458
AML#7	0.41894	0.41487	0.60241
AML#8	0.33128	0.31352	0.35354

ED50: 50% effective dose; ED75: 75% effective dose; ED90: 90% effective dose

### Combination of ST1326 and ABT199 results in synergistic induction of apoptosis and ST1326 prevents up‐regulation of Mcl‐1 induced by ABT199

**Fig. 4:**
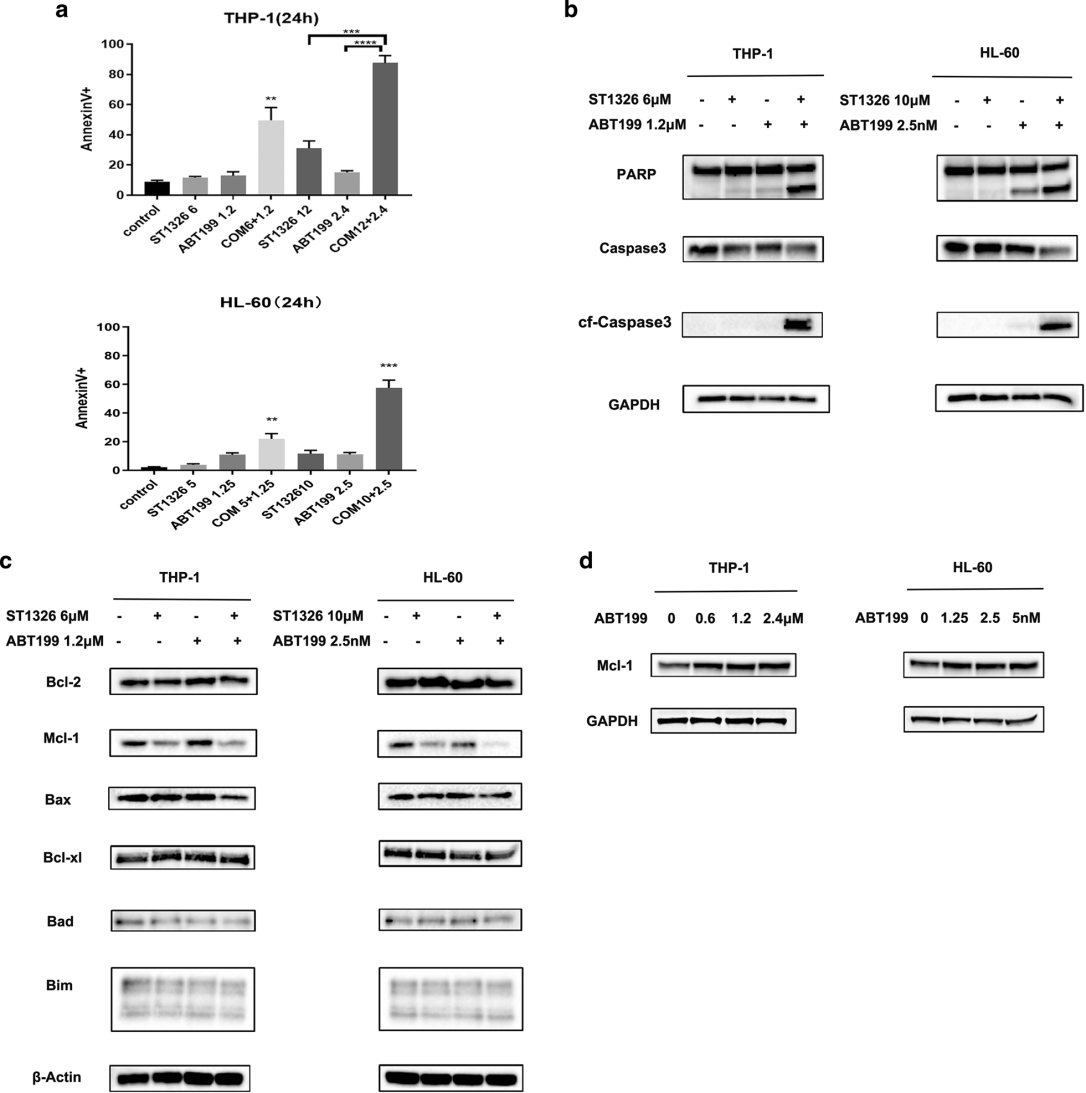
**a** Apoptosis induced by various treatments at 24 h (**P < 0.01, ***P < 0.001, ****P < 0.0001, unpaired t test, combination treatments versus single treatments). **b** THP-1, HL-60 cells were treated with ST1326 and ABT199, alone or combined, for 24 h. Western blot of Caspase-3, cleaved Caspase-3 and PARP-1 in AML cells. **c** THP-1 and HL-60 cells were treated with ST1326 and ABT199, alone or combined, for 24 h. Western blot of Bcl‐2, Bax, Bad, Bcl‐xL, Mcl-1 and Bim in AML cells.**d** THP-1 and HL-60 cells were treated with increasing doses of ABT199 for 24 h. Western blot analysis was conducted for Mcl-1 protein levels

To explore the mechanism of synergistic effect, we treated THP-1 and HL-60 cells with ST1326 and ABT199 at low and high concentrations separately or synergistically for 24 h and then measured cell death by Annexin V/DAPI dual staining. Compared with single agents, combination of ST1326 and ABT199 resulted in a significant increase in apoptosis (Fig. 4a). Moreover, expression of cleaved caspase-3 and cleaved PARP was higher in cells cultured with combinational agents compared with that observed for ST1326 and ABT199 (Fig. 4b). Next, we analyzed the key signaling molecules of relevant Bcl‐2 family proteins, which has great relevant with apoptosis pathway. The levels of Bcl-2, Bax, Bad, Bim and Bcl-xl remained relatively unchanged (Fig. 4c). ST1326 treatment decreased Mcl-1 levels and prevented up-regulation of Mcl-1 induced by ABT199 in THP-1 and HL-60 cell lines (Fig. 4c). Mcl-1 was further up-regulated after short-term exposure to ABT199 (Fig. 4d), which further enhance their resistance to ABT199.

### ST1326 inhibits pGSK3β and pERK to downregulate Mcl-1

**Fig. 5:**
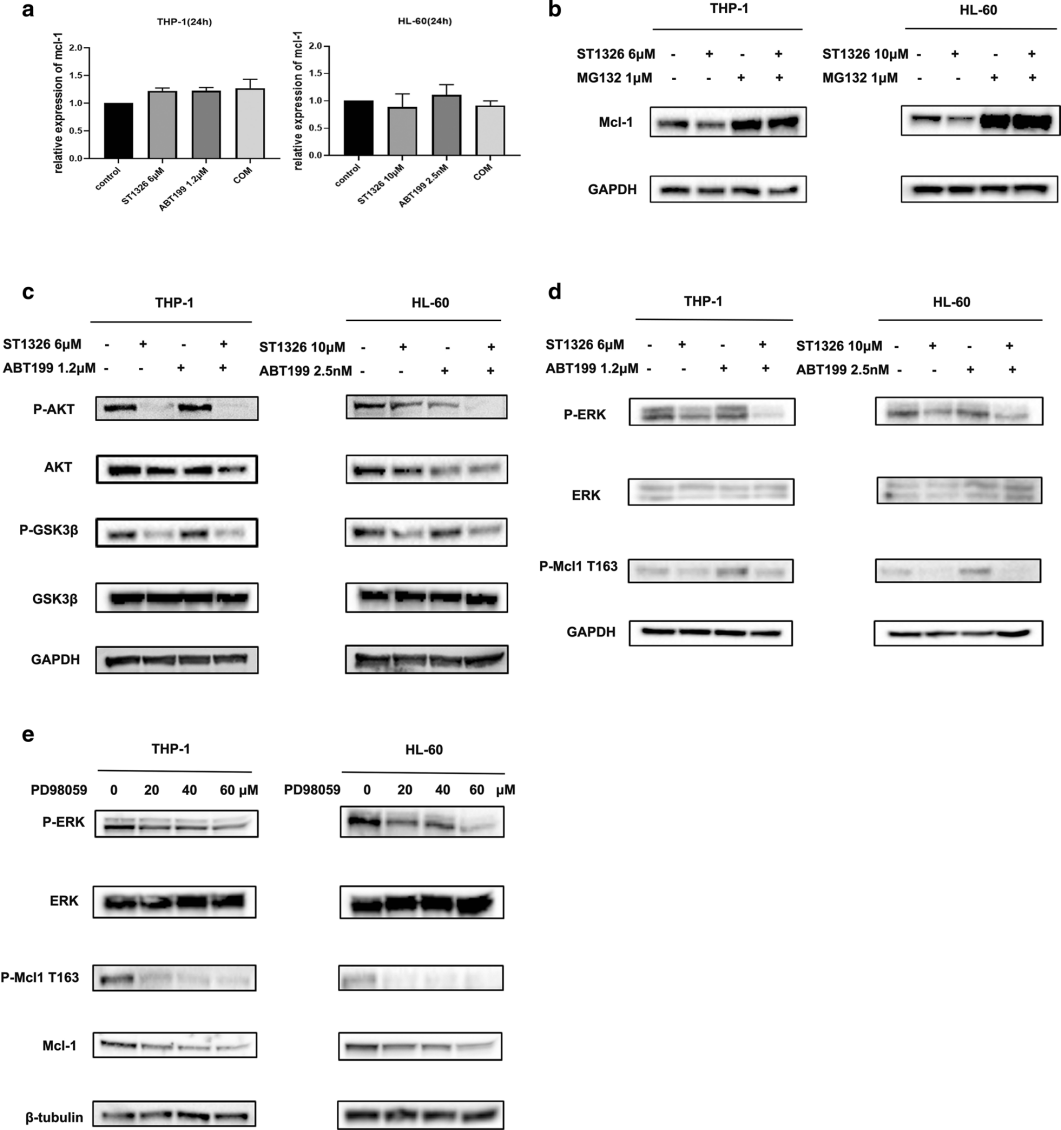
**a** THP-1 and HL-60 cells were treated with ST1326 and ABT199, alone or combined, for 24 h. qPCR analysis of Mcl-1 expression. **b** THP-1 and HL-60 cells were pretreated with 1 μM MG132 (proteasome inhibitor) for 6 h, and then incubated with ST1326 for 18 h. Western blot analysis was conducted for Mcl-1 protein levels. **c** THP-1 and HL-60 cells were treated with ST1326 and ABT199, alone or combined, for 24 h. Western blot analysis was conducted for p-GSK3β, GSK3β, AKT, p-AKT protein levels (**d**). THP-1 and HL-60 cells were treated with ST1326 and ABT199, alone or combined, for 24 h. Western blot analysis was conducted for p-ERK, ERK, p-Mcl1 Thr163 protein levels. **e** THP-1 and HL-60 cells were treated with increasing doses of PD98059 for 24 h. Western blot analysis was conducted for p-ERK, ERK, p-Mcl1 Thr163, Mcl-1 protein levels

To find how ST1326 treatment reduces Mcl-1 protein level, we first performed RT-PCR. Interestingly, Mcl‐1 transcript levels were not decreased in THP-1 and HL-60 cells treated with ST1326 and ABT199, alone or combined for 24 h, indicating a post-transcriptional mechanism (Fig. 5a). Then, a proteasome inhibitor MG132 was used to examine Mcl-1 protein stability. THP-1 and HL-60 cells were treated with ST1326 or MG132 alone or in combination. MG132 pretreatment could suppress ST1326-induced MCL1 down-regulation in THP-1 cells and HL-60 cells (Fig. 5b). These results indicate that the reduction of the Mcl-1 protein levels in AML cells treated with ST1326 might be mediated through proteasome degradation. Previous studies have shown that AKT-mediated GSK3β phosphorylation and MAPK-mediated Mcl-1 phosphorylation are involved in Mcl-1 degradation [18–24]. Therefore, we evaluated the effect of ST1326 on MAPK and AKT activation. ST1326 treatment decreased the phosphorylation of GSK3β at Ser9 and p-AKT (Fig. 5c) and decreased pERK along with pMcl-1Thr163 (Fig. 5d). To further examine the role ERK played in Thr163 phosphorylation, we tested whether ERK inhibition would lead to reduced levels of pMcl-1Thr163. Consistently, inactivation of ERK by PD98059 (ERK/MAPK inhibitor) decreased Mcl-1 expression and its phosphorylation at Thr163 (Fig. 5e).

## Discussion

The Warburg effect proposed the idea that cancer cells rely on aerobic glycolysis even in the presence of abundant oxygen in contrast to normal differentiated cells, which rely primarily on mitochondrial oxidative phosphorylation to generate the energy needed [25]. In addition to changes in glucose metabolism, there is convincing evidence that cancer cells have specific changes in lipid metabolism. Fatty acid oxidation (FAO) is a way to produce adenosine triphosphate, reduced nicotinamide adenine dinucleotide phosphate and acetyl–coenzyme A in cancer cells [5]. As an enzyme of the rate-limiting step of FAO, carnitine palmitoyl transferase1a (CPT1a) plays an important role in cancer metabolic adaptation. Previous study found overexpression of CPT1a was shown in AML than normal BM and PB and high expression of CPT1a is associated with adverse outcomes in AML using public microarray datasets with bioinformatics method [26]. In this study, we agreed that opinion CPT1a is a high risk prognostic factor for AML again with our data of 325 Chinese patients. In parallel, downregulated expression of CPT1a using shRNAs inhibited proliferation of AML cells. However, we found CPT1a mRNA expression was similar between primary AML samples (n = 325) and healthy donors (n = 8). What’s more, we explore the distinctive metabolic patterns associated with CPT1a expression in AML. In high expression of CPT1a group, the decreased expression of the fatty acid, amino acid levels and increasing expression of nucleotide synthesis imply CPT1a expression acts on an oncogene by facilitation of FAO and the utilization of more glucose and amino acids.

Previous study demonstrates that CPT1 can interact with Bcl-2 [16] and the truncated form of the proapoptotic Bcl-2 family member Bid (tBid) decreases CPT1 activity. And overexpression of Bcl-2 antagonizes this effect through direct interaction with CPT1a [17]. Taken together, we hypothesized that down-regulation of CPT1a sensitizes ABT199 to AML cells. The results confirmed our thoughts. What’ more, we discovered for the first time that co-administration of ST1326 and ABT199 resulted in a further increased inhibition of proliferation in AML cell lines and primary patients. Combination of ST1326 and ABT199 resulted in synergistic induction of apoptosis.

Previous study demonstrated that the IC50 of venetoclax was inversely correlated with Bcl-2/Mcl-1 transcript ratio, and over-expression of Bcl-xl or Mcl-1 conferred resistance to venetoclax-induced apoptosis in AML cell lines [27]. In our study, we observed that ST1326 could prevent up-regulation of Mcl-1 induced by ABT199 through proteasome degradation. Previous studies have shown that AKT-mediated GSK3β phosphorylation and MAPK-mediated MCL1 phosphorylation are involved in MCL1 degradation [18–24]. In our study, ST1326 treatment decreased the phosphorylation of GSK3β at Ser9 and p-AKT and decreased pERK along with pMcl-1Thr163.

Preveious study show AML is rarely cured by a single enzyme or pathway, so it is likely that drugs targeting CPT1a will need to be combined with chemotherapy or other targeted drugs to succeed [5]. Meanwhile, in the case of AML, venetoclax is best combined with another agent because resistance seems to develop rather quickly with venetoclax monotherapy [27]. Our study may provide a proper scheme for this dilemma.

## Conclusion

Previous reports demonstrated that CPT1a acts as a potential molecular target in solid tumors and hematologic disease. In our study, CPT1a was found not only have a similar signifcant prognostic use in AML patients in determining overall survival but also involves in multiple pathways. Moreover, we found downregulation of CPT1a sentitized BCL-2 inhibitor ABT199 and CPT1a-selective inhibitor ST1326 combined with ABT199 had a strong synergistic effect to induce apoptosis in AML cells and primary patient blasts for the first time, providing a proper scheme for the problem of ABT199 resistence in AML.

## Supplementary Information

**Additional file 1.** Addiional Tables.

## Data Availability

The datasets generated during the current study are not publicly available due confidentiality for another study but are available from the corresponding author on reasonable request.

## Abbreviations

FAO: Fatty acid oxidation
CPT1a: Carnitine palmitoyltransferase 1a
AML: Acute myeloid leukemia
CLL: Chronic lymphocytic leukemia
Mcl-1: Myeloid cell leukemia sequence 1
