# Supplementary material for: Inhibition of CPT1a as a prognostic marker can synergistically enhance the antileukemic activity of ABT199
Source: J Transl Med. 2021 Apr 29;19:181. doi: 10.1186/s12967-021-02848-9 (PMC8082622; doi:10.1186/s12967-021-02848-9)
Supplement: Supplementary file 1 — Additional file 1. Addiional Tables. [file 12967_2021_2848_MOESM1_ESM.docx]

Table S1 Multivariate analysis of EFS in CN-AML

| Variables | P-value | HR（95%CI） |  |
| --- | --- | --- | --- |
| CPT1A expression | 0.076 | 1.412(0.965,2.066) | |
| age | <0.001 | 1.032(1.021,1.043) | |
| WBC | <0.001 | 1.006(1.004,1.009) | |
| ENL favorable group | <0.001 | 0.366(0.241,0.556) | |
| DNMT3a | 0.05 | 1.534(0.999,2.356) | |
| IDH1 | 0.02 | 1.674(1.086,2.579) | |
| IDH2 | 0.642 | 1.109(0.716,1.719) | |

Table S2 List of cell lines. Related to figures 1,3,4,5.

| Cell line | Morphology | Type | Karyotype and/or mutaions |
| --- | --- | --- | --- |
| HL-60 | promyelocytic | AML, M2 | c-myc+,NRAS |
| THP-1 | monocytic | AML, M5 | MLL-AF9 |
| OCI-AML2 | myelomonocytic | AML, M4 |  |
| OCI-AML3 | myelomonocytic | AML, M4 | NPMc+ |
| KASUMI-1 | myeloblastic | AML, M2 | t(8;21) |
| MV4-11 | myelomonocytic | AML, M5 | FLT3-ITD, MLL-AF9 |
| MOLM-13 | monocytic | AML, M5 | FLT3-ITD, MLL-AF9 |

Table S3-1 Characteristics of CN-AML patients by high and low CPT1A expression in metabolic analysis

|  | Low CPT1a | High CPT1a | p |
| --- | --- | --- | --- |
| n | 6 | 6 |  |
| Male(median [IQR]) | 1 (16.7) | 4 (66.7) | 0.242 |
| age (median [IQR]) | 48.00 [43.25, 52.75] | 39.00 [27.75, 58.50] | 0.522 |
| Blast (median [IQR]) | 40.75 [34.12, 58.25] | 70.50 [52.50, 84.75] | 0.2 |
| WBC (median [IQR]) | 2.30 [2.05, 2.48] | 14.20 [3.33, 50.65] | 0.2 |
| HB (median [IQR]) | 83.50 [56.25, 89.00] | 86.50 [76.75, 97.00] | 0.522 |
| PLT (median [IQR]) | 67.00 [37.50, 166.25] | 111.50 [58.00, 147.00] | 0.749 |
| FAB,n(%) |  |  | 1 |
| M0 | 1 (16.7) | 2 (33.3) |  |
| M1 | 1 (16.7) | 1 (16.7) |  |
| M2 | 2 (33.3) | 1 (16.7) |  |
| M5 | 2 (33.3) | 2 (33.3) |  |
| CEBPA = 1 (%) | 1 (16.7) | 1 (20.0) | 1 |
| FLT3ITD = 1 (%) | 0 ( 0.0) | 2 (33.3) | 0.455 |
| NPM1 = 1 (%) | 1 (16.7) | 2 (40.0) | 0.545 |
| DNMT3A = 1 (%) | 2 (33.3) | 1 (20.0) | 1 |

|  | Low CPT1a | High CTP1a | P value |
| --- | --- | --- | --- |
| n | 11 | 11 |  |
| Age(median [IQR]) | 41.00 [23.50, 54.00] | 48.00 [23.00, 51.50] | 0.792 |
| Male(median [IQR]) | 7 ( 63.6) | 6 ( 54.5) | 1 |
| WBC(median [IQR]) | 21.80 [10.40, 51.25] | 7.60 [3.80, 33.05] | 0.094 |
| HG(median [IQR]) | 72.00 [64.50, 84.50] | 88.00 [73.00, 97.00] | 0.115 |
| PLT(median [IQR]) | 35.00 [29.50, 65.00] | 53.00 [43.00, 97.50] | 0.115 |
| FAB, n (%) |  |  | 0.779 |
| M0 | 0 ( 0.0) | 1 ( 9.1) |  |
| M1 | 1 ( 9.1) | 2 ( 18.2) |  |
| M2 | 4 ( 36.4) | 4 ( 36.4) |  |
| M4 | 1 ( 9.1) | 2 ( 18.2) |  |
| M5 | 4 ( 36.4) | 2 ( 18.2) |  |
| M6 | 1 ( 9.1) | 0 ( 0.0) |  |
| BLAST | 56.00 [46.75, 68.50] | 63.00 [54.50, 83.50] | 0.324 |
| NPM1 = 1 (%) | 3 ( 27.3) | 4 ( 36.4) | 1 |
| CEBPA = 2 (%) | 1 ( 9.1) | 1 ( 9.1) | 1 |
| DNMT3a = 1 (%) | 2 ( 18.2) | 0 ( 0.0) | 0.476 |
| IDH1 = 1 (%) | 2 ( 18.2) | 4 ( 36.4) | 0.635 |
| IDH2 = 1 (%) | 4 ( 36.4) | 0 ( 0.0) | 0.09 |

Table S3-2 Characteristics of CN-AML patients by high and low CPT1a expression in genomic analysis

Table S4 differential gene expression analysis of clinical AML samples associated with CPT1a expression

| gene_short_name | gene_id | logFC | PValue |
| --- | --- | --- | --- |
| TSPAN6 | ENSG00000000003 | 3.94758193 | 0.018039324 |
| YBX2 | ENSG00000006047 | 2.860280896 | 0.022330187 |
| ARHGAP44 | ENSG00000006740 | 4.896722052 | 0.006799621 |
| CD22 | ENSG00000012124 | 1.93999907 | 0.008631535 |
| BIRC3 | ENSG00000023445 | 1.011023003 | 0.010421128 |
| SLAMF7 | ENSG00000026751 | 1.143558947 | 0.034119121 |
| LRRC7 | ENSG00000033122 | 2.745808914 | 0.013265777 |
| MYOM2 | ENSG00000036448 | 1.655465226 | 0.015206974 |
| CDH10 | ENSG00000040731 | 3.440493759 | 0.030943617 |
| ADAMTS6 | ENSG00000049192 | 1.989215264 | 0.007695513 |
| EPN3 | ENSG00000049283 | 4.110014649 | 0.020090161 |
| ENTPD2 | ENSG00000054179 | 4.381564352 | 0.044128747 |
| PITX1 | ENSG00000069011 | 3.54948436 | 0.024965983 |
| FCGR2B | ENSG00000072694 | 1.798147573 | 0.011275526 |
| KCNQ2 | ENSG00000075043 | 4.886888375 | 0.015173505 |
| MAP2 | ENSG00000078018 | 3.681755616 | 0.004503648 |
| ADCY2 | ENSG00000078295 | 3.856941368 | 0.004416503 |
| AFP | ENSG00000081051 | 4.277624878 | 0.027475842 |
| COL4A4 | ENSG00000081052 | 2.277950207 | 0.019932204 |
| CADPS2 | ENSG00000081803 | 2.519768461 | 0.018431606 |
| COL19A1 | ENSG00000082293 | 2.185463632 | 0.006931835 |
| APOB | ENSG00000084674 | 2.300529981 | 0.029972736 |
| SLC26A4 | ENSG00000091137 | 3.831871141 | 0.015676774 |
| TMEM14A | ENSG00000096092 | 1.950683061 | 0.036714153 |
| CRYBB1 | ENSG00000100122 | 3.071471846 | 0.013973518 |
| DHRS2 | ENSG00000100867 | 3.540699988 | 0.018261075 |
| CD40 | ENSG00000101017 | 2.376809403 | 0.00174822 |
| SGK2 | ENSG00000101049 | 1.438682811 | 0.038931088 |
| NKAIN4 | ENSG00000101198 | 5.294255214 | 0.01934866 |
| HTR2A | ENSG00000102468 | 2.774952976 | 0.035687341 |
| CCL22 | ENSG00000102962 | 3.15599054 | 0.04981789 |
| OCA2 | ENSG00000104044 | 3.842584787 | 0.04634972 |
| DKK4 | ENSG00000104371 | 3.650401208 | 0.037964064 |
| LILRB1 | ENSG00000104972 | 1.356241506 | 0.047573753 |
| PLD3 | ENSG00000105223 | 1.125858483 | 0.031359514 |
| PPP1R17 | ENSG00000106341 | 4.216508672 | 0.036107985 |
| GALK1 | ENSG00000108479 | 1.131623517 | 0.026856275 |
| FAM20A | ENSG00000108950 | 2.402462448 | 0.015844117 |
| INPP4B | ENSG00000109452 | 1.393220343 | 0.025686861 |
| CPZ | ENSG00000109625 | 3.735009739 | 0.016119664 |
| CRYAB | ENSG00000109846 | 3.34311129 | 0.048530412 |
| POU2AF1 | ENSG00000110777 | 1.503935285 | 0.041817507 |
| CAPRIN2 | ENSG00000110888 | 1.051660595 | 0.015758579 |
| CCR6 | ENSG00000112486 | 2.118662838 | 0.025748993 |
| GUCA1B | ENSG00000112599 | 1.782368787 | 0.015674537 |
| LPHN2 | ENSG00000117114 | 2.71136598 | 0.025714558 |
| PROX1 | ENSG00000117707 | 3.73622892 | 0.010559777 |
| HMGN3 | ENSG00000118418 | 1.103386034 | 0.009062223 |
| ONECUT2 | ENSG00000119547 | 3.033695673 | 0.048471627 |
| EPCAM | ENSG00000119888 | 3.583578898 | 0.001039766 |
| PLEKHG1 | ENSG00000120278 | 2.149774691 | 0.00318575 |
| SLC10A2 | ENSG00000125255 | 4.811179488 | 0.011451974 |
| C20orf195 | ENSG00000125531 | 2.896096418 | 0.012064174 |
| FLRT3 | ENSG00000125848 | 3.286771553 | 0.0404983 |
| PRRG2 | ENSG00000126460 | 2.476419528 | 0.02031456 |
| RHOJ | ENSG00000126785 | 2.973985998 | 0.045549961 |
| VPREB3 | ENSG00000128218 | 2.359138955 | 0.033494442 |
| AP1M2 | ENSG00000129354 | 4.573412296 | 0.011764698 |
| SLC7A10 | ENSG00000130876 | 3.589400152 | 0.025460851 |
| FCRL2 | ENSG00000132704 | 3.308601475 | 0.000205008 |
| CCNA1 | ENSG00000133101 | 1.749238688 | 0.038603069 |
| EPSTI1 | ENSG00000133106 | 1.588217362 | 0.010399293 |
| TBX3 | ENSG00000135111 | 3.922292304 | 0.030982648 |
| LCA5 | ENSG00000135338 | 1.709085392 | 0.019492385 |
| CHAD | ENSG00000136457 | 3.739306828 | 0.017008989 |
| BLK | ENSG00000136573 | 1.588700239 | 0.006186506 |
| IL10 | ENSG00000136634 | 4.049431435 | 0.004717772 |
| ZFP37 | ENSG00000136866 | 2.213686234 | 0.034678166 |
| CTSV | ENSG00000136943 | 3.818105196 | 0.042918211 |
| LMX1B | ENSG00000136944 | 4.294063298 | 0.032138463 |
| IRF4 | ENSG00000137265 | 1.07140561 | 0.038335953 |
| FOXF2 | ENSG00000137273 | 5.761032373 | 0.005850895 |
| MGARP | ENSG00000137463 | 2.831253882 | 0.006262343 |
| RAB30 | ENSG00000137502 | 1.817781007 | 0.001766907 |
| TRPC6 | ENSG00000137672 | 2.625015556 | 0.049006763 |
| GCOM1 | ENSG00000137878 | 3.591598298 | 0.008169892 |
| IFI44L | ENSG00000137959 | 1.621536756 | 0.027896421 |
| GIPC2 | ENSG00000137960 | 4.008228549 | 0.012791478 |
| CLCA2 | ENSG00000137975 | 3.488513234 | 0.02235791 |
| FGF2 | ENSG00000138685 | 3.779609976 | 0.009280744 |
| SLC25A47 | ENSG00000140107 | 3.282242982 | 0.047561216 |
| PNMT | ENSG00000141744 | 2.769041038 | 0.033469467 |
| FCRL5 | ENSG00000143297 | 1.664562941 | 0.018826208 |
| HHIPL2 | ENSG00000143512 | 2.261199911 | 0.04865216 |
| CYP4V2 | ENSG00000145476 | 1.195612177 | 0.018568779 |
| CRIP3 | ENSG00000146215 | 2.292748991 | 0.028375569 |
| IGFBP3 | ENSG00000146674 | 2.706717352 | 0.005809327 |
| SYTL5 | ENSG00000147041 | 3.880929752 | 0.009198765 |
| DNAJC5B | ENSG00000147570 | 3.985721126 | 0.009491837 |
| SLC26A7 | ENSG00000147606 | 2.948693829 | 0.006033134 |
| LRRC4C | ENSG00000148948 | 3.591019022 | 0.030621092 |
| GRIK4 | ENSG00000149403 | 2.469317944 | 0.019291808 |
| CWC15 | ENSG00000150316 | 1.082167904 | 0.001695402 |
| DLG2 | ENSG00000150672 | 2.206472109 | 0.007733914 |
| PPP1R1C | ENSG00000150722 | 3.028589478 | 0.047724242 |
| TKTL2 | ENSG00000151005 | 4.092559765 | 0.006581184 |
| POU4F2 | ENSG00000151615 | 5.000626373 | 0.025859928 |
| RASGRP3 | ENSG00000152689 | 1.243931893 | 0.019716816 |
| GPR15 | ENSG00000154165 | 2.569889556 | 0.028448828 |
| CNTNAP3B | ENSG00000154529 | 3.784652429 | 0.020779619 |
| NCAM2 | ENSG00000154654 | 3.730999628 | 0.003845409 |
| GRAMD3 | ENSG00000155324 | 1.168715093 | 0.044600338 |
| PPEF2 | ENSG00000156194 | 2.379332494 | 0.02943338 |
| MS4A1 | ENSG00000156738 | 2.133559187 | 0.00571241 |
| KCNJ6 | ENSG00000157542 | 5.124511975 | 0.013009488 |
| NLRP14 | ENSG00000158077 | 4.073857826 | 0.038742002 |
| PFKFB1 | ENSG00000158571 | 2.294144216 | 0.019691303 |
| APOA2 | ENSG00000158874 | 3.86367932 | 0.040325747 |
| SCUBE1 | ENSG00000159307 | 2.357559828 | 0.010361394 |
| CXCR5 | ENSG00000160683 | 2.381326563 | 0.000714651 |
| FBXO27 | ENSG00000161243 | 3.720870814 | 0.024454571 |
| DMKN | ENSG00000161249 | 3.298798463 | 0.03544278 |
| KCNT2 | ENSG00000162687 | 3.176218524 | 0.039087449 |
| FAM71A | ENSG00000162771 | 1.9421221 | 0.020949228 |
| FRZB | ENSG00000162998 | 2.045813221 | 0.048557832 |
| SPATA18 | ENSG00000163071 | 3.334305295 | 0.021721287 |
| FCRL1 | ENSG00000163534 | 1.563574669 | 0.011518184 |
| HEYL | ENSG00000163909 | 1.823018267 | 0.022055884 |
| BSN | ENSG00000164061 | 1.799211134 | 0.034583913 |
| GPX8 | ENSG00000164294 | 2.855362113 | 0.02647268 |
| EBF1 | ENSG00000164330 | 1.555242007 | 0.02979379 |
| IL31RA | ENSG00000164509 | 3.688607272 | 0.028160677 |
| BMPER | ENSG00000164619 | 3.727077715 | 0.001351234 |
| ADCY1 | ENSG00000164742 | 2.935895774 | 0.013214074 |
| FREM1 | ENSG00000164946 | 2.602174852 | 0.034940607 |
| AQP7 | ENSG00000165269 | 2.854068684 | 0.046296047 |
| SLITRK5 | ENSG00000165300 | 3.197965503 | 0.0419558 |
| FAT3 | ENSG00000165323 | 3.151505174 | 0.014534697 |
| GYLTL1B | ENSG00000165905 | 2.16199595 | 0.045114329 |
| CLMP | ENSG00000166250 | 2.177221901 | 0.038150227 |
| CCL23 | ENSG00000167236 | 3.117084443 | 0.010184745 |
| FAM129C | ENSG00000167483 | 1.431381301 | 0.004562633 |
| LAIR2 | ENSG00000167618 | 2.683963626 | 0.006267502 |
| KLK1 | ENSG00000167748 | 3.74271217 | 0.027807672 |
| ACOX2 | ENSG00000168306 | 3.375202047 | 0.022576832 |
| SCN11A | ENSG00000168356 | 2.156236962 | 0.003221861 |
| STXBP6 | ENSG00000168952 | 3.176110071 | 0.004115728 |
| COL4A3 | ENSG00000169031 | 2.960867618 | 0.003898611 |
| SH3TC2 | ENSG00000169247 | 1.576762627 | 0.016739018 |
| P2RY12 | ENSG00000169313 | 3.136346745 | 0.007138211 |
| RSPH10B2 | ENSG00000169402 | 2.979831343 | 0.013548702 |
| SLC38A11 | ENSG00000169507 | 2.736027938 | 0.039940465 |
| HSFY2 | ENSG00000169953 | 4.32432921 | 0.031541927 |
| ANKK1 | ENSG00000170209 | 3.59925723 | 0.035105539 |
| EMX2 | ENSG00000170370 | 7.626458451 | 4.34E-05 |
| BFSP2 | ENSG00000170819 | 4.886746576 | 0.00652073 |
| PLA2G1B | ENSG00000170890 | 2.079477445 | 0.049651163 |
| NPTX1 | ENSG00000171246 | 3.043444519 | 0.044816899 |
| ID4 | ENSG00000172201 | 3.082748811 | 0.020432271 |
| CXCR6 | ENSG00000172215 | 1.408107574 | 0.048507314 |
| B3GALT1 | ENSG00000172318 | 3.741885695 | 0.025351371 |
| TDRD12 | ENSG00000173809 | 6.002150608 | 0.002681984 |
| CHDC2 | ENSG00000176034 | 2.78861208 | 0.029807682 |
| CD19 | ENSG00000177455 | 2.241545295 | 0.001988472 |
| FAM26E | ENSG00000178033 | 2.804597833 | 0.049318727 |
| TUBAL3 | ENSG00000178462 | 3.925640714 | 0.001226012 |
| APOLD1 | ENSG00000178878 | 1.373494723 | 0.007758597 |
| GGN | ENSG00000179168 | 3.256583706 | 0.048799403 |
| DNAJB8 | ENSG00000179407 | 4.248442627 | 0.034111582 |
| GOLGA8J | ENSG00000179938 | 1.819227023 | 0.033926473 |
| SKIDA1 | ENSG00000180592 | 2.605676159 | 0.007921186 |
| SHISA2 | ENSG00000180730 | 4.437720149 | 0.002717821 |
| CAPS2 | ENSG00000180881 | 1.49798179 | 0.046313544 |
| FAM153B | ENSG00000182230 | 2.028867361 | 0.041913516 |
| IZUMO1 | ENSG00000182264 | 3.574037459 | 0.013890669 |
| FAM156A | ENSG00000182646 | 3.307566866 | 0.000729639 |
| CADM1 | ENSG00000182985 | 2.936404418 | 0.006513713 |
| CTNNA3 | ENSG00000183230 | 3.996661217 | 0.029404474 |
| OR51B4 | ENSG00000183251 | 3.670991337 | 0.010639706 |
| GBP6 | ENSG00000183347 | 2.773025217 | 0.048879379 |
| PTPN20B | ENSG00000183675 | 3.608790623 | 0.011409729 |
| HIST2H4A | ENSG00000183941 | 1.789066139 | 0.011313884 |
| OR2T10 | ENSG00000184022 | 4.522885339 | 0.038927429 |
| RGPD2 | ENSG00000185304 | 2.099312441 | 0.006936694 |
| FFAR3 | ENSG00000185897 | 2.919491987 | 0.015551796 |
| CCDC30 | ENSG00000186409 | 1.008203772 | 0.036701304 |
| ERAS | ENSG00000187682 | 2.724398755 | 0.038004979 |
| TMEM72 | ENSG00000187783 | 3.56817045 | 0.041623325 |
| OR7D2 | ENSG00000188000 | 3.030792059 | 0.04188261 |
| CTD-3088G3.8 | ENSG00000188897 | 1.31059294 | 0.034236692 |
| PAX5 | ENSG00000196092 | 1.399220913 | 0.006153494 |
| S100A2 | ENSG00000196754 | 2.460672334 | 0.044698069 |
| LYPD2 | ENSG00000197353 | 5.002967105 | 0.018405031 |
| GOLGA6L9 | ENSG00000197978 | 1.893550326 | 0.022746338 |
| OR2T6 | ENSG00000198104 | 4.550331963 | 0.047909529 |
| RTP2 | ENSG00000198471 | 3.918126392 | 0.043260012 |
| OR2M2 | ENSG00000198601 | 3.33660019 | 0.032773202 |
| RYR3 | ENSG00000198838 | 2.675717657 | 0.002114146 |
| MT-ND3 | ENSG00000198840 | 7.004540336 | 0.000696787 |
| CES1 | ENSG00000198848 | 2.619746108 | 0.039236151 |
| RORB | ENSG00000198963 | 4.251237522 | 0.002358819 |
| AGAP10 | ENSG00000204172 | 1.58429712 | 0.016018938 |
| CXorf30 | ENSG00000205081 | 3.503381223 | 0.017381278 |
| CCNI2 | ENSG00000205089 | 2.795908581 | 0.044626947 |
| SCGB2B2 | ENSG00000205209 | 2.520606799 | 0.011265081 |
| EXOC3L4 | ENSG00000205436 | 4.573804884 | 0.000413218 |
| AC006435.1 | ENSG00000205821 | 3.417289887 | 0.039939854 |
| ONECUT3 | ENSG00000205922 | 4.253620637 | 0.034557833 |
| OLIG2 | ENSG00000205927 | 4.660309437 | 0.005363162 |
| DOK6 | ENSG00000206052 | 3.775429998 | 0.009289851 |
| TAS2R13 | ENSG00000212128 | 1.78941997 | 0.014135965 |
| C12orf74 | ENSG00000214215 | 3.786091847 | 0.010088332 |
| ARHGEF33 | ENSG00000214694 | 2.912525045 | 0.004976716 |
| ISPD | ENSG00000214960 | 1.160707371 | 0.043467739 |
| GCGR | ENSG00000215644 | 3.830416396 | 0.046270519 |
| LILRA4 | ENSG00000239961 | 3.265741488 | 0.002184346 |
| LRRD1 | ENSG00000240720 | 2.588303372 | 0.026607382 |
| TCP10L | ENSG00000242220 | 3.210425259 | 0.005686911 |
| PEG10 | ENSG00000242265 | 3.086338207 | 0.00369652 |
| UGT1A10 | ENSG00000242515 | 4.190395832 | 0.029960718 |
| TNFSF12-TNFSF13 | ENSG00000248871 | 5.171313798 | 0.006550673 |
| RP11-514O12.4 | ENSG00000249141 | 2.212180985 | 0.03719704 |
| AP000304.12 | ENSG00000249209 | 4.770058877 | 0.028134076 |
| FOXD1 | ENSG00000251493 | 2.575682543 | 0.04783852 |
| RP11-108K14.8 | ENSG00000254536 | 5.617211403 | 0.008386924 |
| SDHD | ENSG00000255292 | 4.162291278 | 0.004272192 |
| RP11-831H9.11 | ENSG00000255432 | 6.074272921 | 0.001784225 |
| MTRNR2L8 | ENSG00000255823 | 7.621802583 | 0.000679485 |
| CTD-3074O7.11 | ENSG00000256349 | 5.643096001 | 0.015739876 |
| RP11-1070N10.3 | ENSG00000258572 | 1.936230251 | 0.037383474 |
| TVP23C-CDRT4 | ENSG00000259024 | 2.151747668 | 0.037900216 |
| RP11-89K11.1 | ENSG00000259658 | 2.465668951 | 0.003411396 |
| RP11-599B13.6 | ENSG00000263620 | 5.680163968 | 0.017861303 |
| RP11-162A12.2 | ENSG00000264278 | 2.936226059 | 0.018299069 |
| RP11-219A15.1 | ENSG00000266302 | 4.70762402 | 0.025824687 |
| IER3IP1 | ENSG00000267228 | 5.174839277 | 0.002296741 |
| AC005779.2 | ENSG00000267545 | 2.880413454 | 0.013645019 |
| SMIM17 | ENSG00000268182 | 2.757116018 | 0.028364395 |
| MTRNR2L12 | ENSG00000269028 | 6.115386873 | 0.006244244 |
| SPIB | ENSG00000269404 | 1.845739777 | 0.012091083 |
| TSNAX-DISC1 | ENSG00000270106 | 5.532216467 | 0.007351298 |
| MTRNR2L2 | ENSG00000271043 | 6.709065829 | 0.00068752 |
| TM4SF19 | ENSG00000273331 | 4.020764452 | 0.012646058 |
| SEMA3F | ENSG00000001617 | -2.920141754 | 0.012539653 |
| CACNG3 | ENSG00000006116 | -4.228679597 | 0.020663238 |
| GTF2IRD1 | ENSG00000006704 | -2.001182842 | 0.038065163 |
| PAX6 | ENSG00000007372 | -4.738520433 | 0.010009668 |
| E2F2 | ENSG00000007968 | -1.070872118 | 0.046115678 |
| PGLYRP1 | ENSG00000008438 | -4.36392355 | 6.55E-05 |
| MMP25 | ENSG00000008516 | -2.355502722 | 7.34E-05 |
| CD9 | ENSG00000010278 | -1.647221644 | 0.023239118 |
| DPF1 | ENSG00000011332 | -2.740454145 | 0.017724439 |
| QPCTL | ENSG00000011478 | -1.199443247 | 0.018380742 |
| LTF | ENSG00000012223 | -3.095015841 | 8.97E-05 |
| ALOX5 | ENSG00000012779 | -1.61825009 | 0.004799775 |
| SLC25A39 | ENSG00000013306 | -1.063134555 | 0.037294449 |
| MAMLD1 | ENSG00000013619 | -1.706901377 | 0.011702889 |
| ISL1 | ENSG00000016082 | -4.742070468 | 0.031337862 |
| IL20RA | ENSG00000016402 | -5.665295543 | 0.013075673 |
| VSIG2 | ENSG00000019102 | -4.563543927 | 0.003787649 |
| FHL1 | ENSG00000022267 | -1.876036016 | 0.029507403 |
| SLC45A4 | ENSG00000022567 | -1.269152987 | 0.004826163 |
| DAPK2 | ENSG00000035664 | -1.588349353 | 0.006487845 |
| CYP46A1 | ENSG00000036530 | -4.391110961 | 0.004201518 |
| HOXC8 | ENSG00000037965 | -4.911605031 | 0.024530103 |
| MYO16 | ENSG00000041515 | -2.700246056 | 0.033339619 |
| GPM6B | ENSG00000046653 | -2.625675514 | 0.021675958 |
| PREX2 | ENSG00000046889 | -2.713044089 | 0.020880708 |
| ELN | ENSG00000049540 | -3.318964337 | 0.036067496 |
| DKK3 | ENSG00000050165 | -4.740759059 | 0.006946212 |
| PSD | ENSG00000059915 | -2.607190229 | 0.005417578 |
| ERBB3 | ENSG00000065361 | -6.093324382 | 0.000254062 |
| COL17A1 | ENSG00000065618 | -3.862932765 | 0.001367743 |
| MPPED2 | ENSG00000066382 | -6.019395319 | 0.002803326 |
| CLDN18 | ENSG00000066405 | -7.965825446 | 0.001107913 |
| FGFR2 | ENSG00000066468 | -3.291755044 | 0.003349286 |
| STAG3 | ENSG00000066923 | -1.612854736 | 0.044623541 |
| HYAL2 | ENSG00000068001 | -1.279928275 | 0.014757266 |
| SDK2 | ENSG00000069188 | -1.778247312 | 0.020679885 |
| LRP6 | ENSG00000070018 | -3.231303225 | 0.00234651 |
| EPHA8 | ENSG00000070886 | -3.199835916 | 0.048546525 |
| LPHN1 | ENSG00000072071 | -2.36655563 | 0.003277342 |
| EVC | ENSG00000072840 | -2.763098626 | 0.025524147 |
| FERMT2 | ENSG00000073712 | -2.231398738 | 0.033894975 |
| PTGS2 | ENSG00000073756 | -1.190393317 | 0.04121565 |
| FSCN1 | ENSG00000075618 | -1.469343269 | 0.014215387 |
| PLXNA2 | ENSG00000076356 | -1.837175786 | 0.015720297 |
| ICAM3 | ENSG00000076662 | -1.000598974 | 0.000276806 |
| NFKB2 | ENSG00000077150 | -1.044926177 | 0.034748091 |
| FGFR1 | ENSG00000077782 | -1.484710136 | 0.0189653 |
| SMC1B | ENSG00000077935 | -4.876266826 | 0.002948596 |
| CST7 | ENSG00000077984 | -1.595609969 | 0.002325721 |
| NEBL | ENSG00000078114 | -2.617191869 | 0.026583793 |
| SYNJ2 | ENSG00000078269 | -1.386809365 | 0.003225404 |
| RUNX1T1 | ENSG00000079102 | -6.736867795 | 6.62E-05 |
| CEACAM1 | ENSG00000079385 | -2.815940808 | 0.000218106 |
| MAGI3 | ENSG00000081026 | -3.346856258 | 0.000569234 |
| PCDHGA2 | ENSG00000081853 | -2.713243705 | 0.025054617 |
| ATP8B1 | ENSG00000081923 | -6.074895227 | 2.28E-05 |
| MECOM | ENSG00000085276 | -4.866471111 | 0.00243386 |
| CPNE3 | ENSG00000085719 | -1.316358856 | 0.00036293 |
| CEACAM6 | ENSG00000086548 | -3.888458508 | 1.61E-05 |
| ACHE | ENSG00000087085 | -2.089119219 | 0.044136313 |
| SMOX | ENSG00000088826 | -1.242112934 | 0.016515841 |
| TESC | ENSG00000088992 | -1.122948675 | 0.028370085 |
| FXYD3 | ENSG00000089356 | -2.984852561 | 0.048768244 |
| ICAM1 | ENSG00000090339 | -1.049705654 | 0.018745241 |
| NLRC4 | ENSG00000091106 | -1.715032656 | 0.01405445 |
| ANGPT2 | ENSG00000091879 | -4.457906319 | 0.006120004 |
| CD200 | ENSG00000091972 | -1.909113247 | 0.014575567 |
| CEBPE | ENSG00000092067 | -1.846900919 | 0.019852161 |
| SLC7A8 | ENSG00000092068 | -1.762342464 | 0.032024007 |
| DFNB31 | ENSG00000095397 | -1.239701944 | 0.018151868 |
| TREM2 | ENSG00000095970 | -4.582225807 | 0.01018151 |
| CRISP3 | ENSG00000096006 | -2.493512567 | 0.014440199 |
| PGC | ENSG00000096088 | -8.402138844 | 0.000382273 |
| SCD | ENSG00000099194 | -1.750292564 | 0.000340459 |
| NRP1 | ENSG00000099250 | -2.061307748 | 0.003828752 |
| PRTFDC1 | ENSG00000099256 | -2.566050479 | 0.022482933 |
| KDELR3 | ENSG00000100196 | -3.808959787 | 0.023074316 |
| DMC1 | ENSG00000100206 | -1.923025265 | 0.014171938 |
| TIMP3 | ENSG00000100234 | -2.058261572 | 0.005427923 |
| BIK | ENSG00000100290 | -1.838412598 | 0.042820219 |
| TSPO | ENSG00000100300 | -1.117916696 | 0.027892158 |
| ABHD4 | ENSG00000100439 | -1.178187756 | 0.006807652 |
| NFATC4 | ENSG00000100968 | -3.205405115 | 0.016072394 |
| MMP9 | ENSG00000100985 | -2.154617025 | 0.003283918 |
| PLCB4 | ENSG00000101333 | -2.542034303 | 0.019938424 |
| BPI | ENSG00000101425 | -2.70111468 | 0.000427873 |
| BMX | ENSG00000102010 | -1.990247678 | 0.046807646 |
| MLNR | ENSG00000102539 | -2.958769043 | 0.006030225 |
| KLF5 | ENSG00000102554 | -1.872178564 | 0.011391612 |
| RGCC | ENSG00000102760 | -1.238292871 | 0.014904625 |
| MSLN | ENSG00000102854 | -2.650597311 | 0.045477045 |
| PLLP | ENSG00000102934 | -4.224337006 | 0.042882185 |
| ESRP2 | ENSG00000103067 | -4.814937195 | 0.002959505 |
| NECAB2 | ENSG00000103154 | -4.417403416 | 0.005006388 |
| SLC7A5 | ENSG00000103257 | -1.10857194 | 0.038191309 |
| CORO2B | ENSG00000103647 | -2.252010266 | 0.04784623 |
| FAM189A1 | ENSG00000104059 | -7.181301421 | 0.000926665 |
| NCALD | ENSG00000104490 | -1.718188834 | 0.017882686 |
| RELB | ENSG00000104856 | -1.693339526 | 0.004337897 |
| AMH | ENSG00000104899 | -2.270797822 | 0.017929287 |
| RETN | ENSG00000104918 | -2.050188371 | 0.032600486 |
| CLC | ENSG00000105205 | -2.390267024 | 0.014554626 |
| CLIP3 | ENSG00000105270 | -2.708423769 | 0.007320698 |
| SLC1A5 | ENSG00000105281 | -1.072662492 | 0.015224799 |
| MYH14 | ENSG00000105357 | -4.074831012 | 0.047382919 |
| ATP4A | ENSG00000105675 | -8.976398123 | 0.000336498 |
| TFPI2 | ENSG00000105825 | -7.330833767 | 0.000975883 |
| NPTX2 | ENSG00000106236 | -3.382666684 | 0.023944251 |
| SERPINE1 | ENSG00000106366 | -1.996857619 | 0.017996862 |
| AHR | ENSG00000106546 | -1.133080359 | 0.030636138 |
| MEGF9 | ENSG00000106780 | -1.059716765 | 0.007679232 |
| CORO2A | ENSG00000106789 | -1.386171604 | 0.009677301 |
| CA9 | ENSG00000107159 | -4.839553099 | 0.018205509 |
| MPDZ | ENSG00000107186 | -4.863343722 | 0.000569618 |
| LHX3 | ENSG00000107187 | -2.832908282 | 0.028383364 |
| GATA3 | ENSG00000107485 | -3.36040026 | 0.001849767 |
| PALD1 | ENSG00000107719 | -1.500186838 | 0.02456029 |
| UNC5B | ENSG00000107731 | -1.519574963 | 0.038389435 |
| SH3PXD2A | ENSG00000107957 | -1.092849779 | 0.019533002 |
| CYP2C18 | ENSG00000108242 | -5.040276242 | 0.010414524 |
| RUNDC3A | ENSG00000108309 | -2.117554139 | 0.038613014 |
| RASD1 | ENSG00000108551 | -1.640850236 | 0.037212287 |
| ALOX12 | ENSG00000108839 | -2.866863496 | 0.01023381 |
| CDR2L | ENSG00000109089 | -3.314493469 | 0.011106104 |
| MAPK10 | ENSG00000109339 | -3.990104459 | 0.006851682 |
| AADAT | ENSG00000109576 | -3.617374686 | 0.003306701 |
| BST1 | ENSG00000109743 | -1.36969725 | 0.018155962 |
| DTX4 | ENSG00000110042 | -1.260538056 | 0.019635436 |
| CCKBR | ENSG00000110148 | -4.205937086 | 0.047446609 |
| GALNT18 | ENSG00000110328 | -3.307745537 | 0.004237272 |
| ELMOD1 | ENSG00000110675 | -5.020775946 | 0.018158398 |
| KRT18 | ENSG00000111057 | -2.18695183 | 0.013047878 |
| TRPV4 | ENSG00000111199 | -2.761574827 | 0.049992223 |
| CUX2 | ENSG00000111249 | -3.607646949 | 0.017128693 |
| MANSC1 | ENSG00000111261 | -2.425175578 | 0.016318897 |
| ENDOU | ENSG00000111405 | -3.016785168 | 0.038223721 |
| FRK | ENSG00000111816 | -3.487965342 | 0.026413829 |
| SLC26A8 | ENSG00000112053 | -2.179122565 | 0.003169312 |
| GLP1R | ENSG00000112164 | -5.903132906 | 0.005428267 |
| GPR63 | ENSG00000112218 | -3.841632516 | 0.011661853 |
| VNN2 | ENSG00000112303 | -1.394518274 | 0.017372449 |
| TBX18 | ENSG00000112837 | -5.965751763 | 0.002780324 |
| LOX | ENSG00000113083 | -4.256464416 | 0.000960633 |
| SPARC | ENSG00000113140 | -1.282344123 | 0.045897412 |
| BTNL8 | ENSG00000113303 | -2.657454704 | 0.03189976 |
| UPK1B | ENSG00000114638 | -4.990193117 | 0.01840838 |
| EFCC1 | ENSG00000114654 | -4.402011087 | 0.001612947 |
| KIAA1257 | ENSG00000114656 | -2.954880192 | 0.002505005 |
| ARHGEF26 | ENSG00000114790 | -4.140612982 | 0.003155952 |
| TFCP2L1 | ENSG00000115112 | -5.675670342 | 0.000769001 |
| OTOF | ENSG00000115155 | -6.543049612 | 9.34E-06 |
| TANC1 | ENSG00000115183 | -2.266084309 | 0.008698689 |
| GCA | ENSG00000115271 | -1.319501488 | 0.001835559 |
| FN1 | ENSG00000115414 | -2.634252886 | 0.004371597 |
| IGFBP2 | ENSG00000115457 | -3.186238654 | 0.000224544 |
| MLPH | ENSG00000115648 | -4.975990119 | 0.005231558 |
| QPCT | ENSG00000115828 | -2.495924065 | 0.002791566 |
| EPAS1 | ENSG00000116016 | -1.585933667 | 0.025673361 |
| PARD3B | ENSG00000116117 | -2.817101805 | 0.026339227 |
| PADI2 | ENSG00000117115 | -2.798792133 | 4.89E-06 |
| KIF17 | ENSG00000117245 | -3.194531396 | 0.01259952 |
| CNN3 | ENSG00000117519 | -2.679798386 | 0.019817963 |
| A4GNT | ENSG00000118017 | -5.51453126 | 0.011874277 |
| MMP8 | ENSG00000118113 | -3.923911116 | 2.18E-06 |
| ARG1 | ENSG00000118520 | -2.178801806 | 0.000877007 |
| PPL | ENSG00000118898 | -4.663051479 | 0.001289395 |
| TRPM6 | ENSG00000119121 | -2.658130035 | 0.004374751 |
| IRF2BPL | ENSG00000119669 | -1.070789799 | 0.018770243 |
| CYSTM1 | ENSG00000120306 | -1.723588533 | 0.024718615 |
| PCDHB8 | ENSG00000120322 | -5.364273709 | 0.001608147 |
| TP53AIP1 | ENSG00000120471 | -4.566247378 | 0.002451565 |
| KIAA1217 | ENSG00000120549 | -3.030223231 | 0.008705176 |
| MRC1 | ENSG00000120586 | -2.389922173 | 0.038199972 |
| IQSEC3 | ENSG00000120645 | -2.225746669 | 0.00533792 |
| EPX | ENSG00000121053 | -2.983309317 | 0.010992192 |
| TBX2 | ENSG00000121068 | -3.091551316 | 0.032665828 |
| LRAT | ENSG00000121207 | -4.239559173 | 0.021975732 |
| PLBD1 | ENSG00000121316 | -1.620923174 | 0.007898352 |
| GJB6 | ENSG00000121742 | -4.616739248 | 0.000186101 |
| INHBA | ENSG00000122641 | -1.521081805 | 0.04588748 |
| TWIST1 | ENSG00000122691 | -5.981583176 | 6.53E-05 |
| NUDT10 | ENSG00000122824 | -3.889097141 | 0.020170441 |
| BHLHE41 | ENSG00000123095 | -1.891241798 | 0.025058874 |
| NECAB1 | ENSG00000123119 | -4.237904949 | 0.007116541 |
| G0S2 | ENSG00000123689 | -1.981463754 | 0.018397198 |
| SLPI | ENSG00000124107 | -2.303591627 | 0.0070678 |
| VAMP7 | ENSG00000124333 | -1.740839835 | 0.027260485 |
| HIF3A | ENSG00000124440 | -4.371440376 | 0.01118439 |
| CEACAM8 | ENSG00000124469 | -3.20196633 | 3.28E-05 |
| EREG | ENSG00000124882 | -2.082408781 | 0.006352839 |
| SSUH2 | ENSG00000125046 | -4.009928311 | 0.045823022 |
| HS3ST3B1 | ENSG00000125430 | -2.21128024 | 0.000727555 |
| GRIA3 | ENSG00000125675 | -4.199329404 | 0.011262144 |
| CD93 | ENSG00000125810 | -1.170690837 | 0.038975464 |
| CHURC1-FNTB | ENSG00000125954 | -3.315425479 | 0.039297799 |
| CFP | ENSG00000126759 | -1.054330676 | 0.023673412 |
| TSPAN8 | ENSG00000127324 | -5.838096311 | 0.010783309 |
| PTPRB | ENSG00000127329 | -3.215867459 | 0.005568742 |
| KRT17 | ENSG00000128422 | -3.119950242 | 0.04201121 |
| CPA4 | ENSG00000128510 | -7.407801716 | 0.001622348 |
| FLNC | ENSG00000128591 | -4.552567012 | 0.000405148 |
| LRRC4 | ENSG00000128594 | -1.728326795 | 0.004354113 |
| GDF2 | ENSG00000128802 | -4.205935883 | 0.047428186 |
| LOXL1 | ENSG00000129038 | -2.318903477 | 0.036964599 |
| FGF13 | ENSG00000129682 | -5.24063783 | 6.90E-05 |
| PVRL2 | ENSG00000130202 | -1.197076605 | 0.048953754 |
| LRCH2 | ENSG00000130224 | -3.596873235 | 0.027572167 |
| HELZ2 | ENSG00000130589 | -1.093518488 | 0.046258326 |
| HBZ | ENSG00000130656 | -3.636433095 | 0.017914086 |
| CASZ1 | ENSG00000130940 | -1.128349315 | 0.033699594 |
| EPS8L1 | ENSG00000131037 | -2.019946221 | 0.037564664 |
| EMR3 | ENSG00000131355 | -2.118247931 | 0.016524036 |
| SH3BP5 | ENSG00000131370 | -1.713060867 | 0.006982706 |
| TUBG1 | ENSG00000131462 | -1.019954077 | 0.045886393 |
| BARX1 | ENSG00000131668 | -4.264020854 | 0.036720686 |
| DDC | ENSG00000132437 | -4.695277789 | 0.013103265 |
| RAB25 | ENSG00000132698 | -3.59440798 | 0.026992688 |
| CHI3L1 | ENSG00000133048 | -4.375026929 | 1.88E-05 |
| TMCC2 | ENSG00000133069 | -1.701487796 | 0.015895072 |
| BEX1 | ENSG00000133169 | -4.060137213 | 0.001007642 |
| PDZD2 | ENSG00000133401 | -1.82514506 | 0.016943857 |
| UNC79 | ENSG00000133958 | -4.189077007 | 0.001573067 |
| IRAK2 | ENSG00000134070 | -2.164205001 | 0.001325312 |
| SYT6 | ENSG00000134207 | -3.203754384 | 0.025905061 |
| CHIA | ENSG00000134216 | -5.928275928 | 0.009755249 |
| NAV1 | ENSG00000134369 | -2.66301891 | 4.89E-06 |
| HRH4 | ENSG00000134489 | -1.783937424 | 0.041427124 |
| GIF | ENSG00000134812 | -7.678106686 | 0.001503763 |
| TCN1 | ENSG00000134827 | -1.793712555 | 0.023323535 |
| COL4A2 | ENSG00000134871 | -3.180790495 | 0.008022928 |
| CCNJL | ENSG00000135083 | -4.45104194 | 0.000945919 |
| MRAP2 | ENSG00000135324 | -3.637241624 | 0.035623809 |
| DYSF | ENSG00000135636 | -1.420475896 | 0.011937897 |
| KCNMB4 | ENSG00000135643 | -1.688792863 | 0.040563546 |
| EGLN1 | ENSG00000135766 | -1.072888974 | 0.008414247 |
| CKAP4 | ENSG00000136026 | -1.421732869 | 0.006039872 |
| SLC41A2 | ENSG00000136052 | -1.694847578 | 0.020400089 |
| TM6SF1 | ENSG00000136404 | -2.572270167 | 0.00426011 |
| IL33 | ENSG00000137033 | -3.397250075 | 0.016397893 |
| TMEM63B | ENSG00000137216 | -1.099109127 | 0.011485043 |
| SULF1 | ENSG00000137573 | -4.233940968 | 0.011977961 |
| TMPRSS4 | ENSG00000137648 | -3.663978255 | 0.031055035 |
| POU2F3 | ENSG00000137709 | -4.652587177 | 0.005689371 |
| THBS1 | ENSG00000137801 | -3.133901596 | 0.000364122 |
| DUOX1 | ENSG00000137857 | -4.121673399 | 0.014116401 |
| CYP2C9 | ENSG00000138109 | -4.934261097 | 0.013289412 |
| LOXL4 | ENSG00000138131 | -2.246301053 | 0.044264459 |
| CEP55 | ENSG00000138180 | -1.431313048 | 0.016176945 |
| ENTPD1 | ENSG00000138185 | -1.242930369 | 0.022031988 |
| OIT3 | ENSG00000138315 | -4.628207668 | 0.015155625 |
| AGPAT9 | ENSG00000138678 | -1.296590184 | 0.017709475 |
| PRDM5 | ENSG00000138738 | -2.15544998 | 0.039190153 |
| ANXA3 | ENSG00000138772 | -3.08650621 | 3.59E-05 |
| B4GALNT3 | ENSG00000139044 | -3.282186359 | 0.002057452 |
| PIK3C2G | ENSG00000139144 | -4.742069946 | 0.031328 |
| GPR84 | ENSG00000139572 | -2.150641931 | 0.003692432 |
| NPFF | ENSG00000139574 | -2.833466643 | 0.046067809 |
| RAB15 | ENSG00000139998 | -2.638608416 | 0.020640435 |
| DUOX2 | ENSG00000140279 | -3.023496539 | 0.01254992 |
| BCL2A1 | ENSG00000140379 | -1.495282879 | 0.002975864 |
| MESDC1 | ENSG00000140406 | -1.363478079 | 0.004464971 |
| IGF1R | ENSG00000140443 | -1.46836131 | 0.001122218 |
| ARMC5 | ENSG00000140691 | -1.370448024 | 0.014431754 |
| MYLK3 | ENSG00000140795 | -2.202676034 | 0.038648234 |
| ZFHX3 | ENSG00000140836 | -2.094810457 | 0.000209704 |
| KIFC3 | ENSG00000140859 | -2.291731659 | 0.011174244 |
| DPEP3 | ENSG00000141096 | -3.777067608 | 0.014465488 |
| PIK3R5 | ENSG00000141506 | -1.205827808 | 0.010398718 |
| CBLN2 | ENSG00000141668 | -2.666315007 | 0.0244916 |
| IGFBP4 | ENSG00000141753 | -2.584799696 | 0.003589766 |
| hsa-mir-1199 | ENSG00000141854 | -2.321012902 | 0.030406451 |
| LMTK3 | ENSG00000142235 | -1.986157856 | 0.037724482 |
| CACNG8 | ENSG00000142408 | -2.958443542 | 0.038124247 |
| C1orf222 | ENSG00000142609 | -3.531361438 | 0.03141495 |
| FHAD1 | ENSG00000142621 | -3.106756415 | 0.021866119 |
| ZNF697 | ENSG00000143067 | -1.195670443 | 0.03477026 |
| PVRL4 | ENSG00000143217 | -3.457707296 | 0.02732111 |
| S100A8 | ENSG00000143546 | -2.703220672 | 3.58E-05 |
| S100A7 | ENSG00000143556 | -5.20594704 | 0.019858858 |
| GALNT13 | ENSG00000144278 | -4.684193765 | 0.043176551 |
| FAM171B | ENSG00000144369 | -3.336335762 | 0.004383128 |
| FAM198A | ENSG00000144649 | -2.56302819 | 0.010266313 |
| IL17RD | ENSG00000144730 | -3.664934767 | 0.02030751 |
| TRPC1 | ENSG00000144935 | -2.197570233 | 0.039206456 |
| CORIN | ENSG00000145244 | -2.666608321 | 0.04319647 |
| DDIT4L | ENSG00000145358 | -2.951945535 | 0.03318571 |
| FAM105A | ENSG00000145569 | -1.218855235 | 0.033610018 |
| SLC25A48 | ENSG00000145832 | -4.249947798 | 0.023980926 |
| GABRB2 | ENSG00000145864 | -4.233352303 | 0.012342719 |
| N4BP3 | ENSG00000145911 | -2.359325623 | 0.013898477 |
| SLC2A12 | ENSG00000146411 | -4.521569971 | 0.009484863 |
| SLC16A2 | ENSG00000147100 | -3.996813748 | 0.011264475 |
| IGSF1 | ENSG00000147255 | -4.484084178 | 0.024369521 |
| ATP6V0D2 | ENSG00000147614 | -4.173455301 | 0.00715154 |
| ZNF462 | ENSG00000148143 | -3.233423121 | 0.005848328 |
| GSN | ENSG00000148180 | -1.02234015 | 0.009708006 |
| CRB2 | ENSG00000148204 | -4.046101428 | 0.007617575 |
| LCN2 | ENSG00000148346 | -2.444151627 | 0.007149604 |
| HMCN2 | ENSG00000148357 | -3.530264849 | 0.04173979 |
| FAM171A1 | ENSG00000148468 | -6.08053663 | 2.04E-05 |
| TMEM236 | ENSG00000148483 | -4.431971908 | 0.017203938 |
| ADM | ENSG00000148926 | -1.295681988 | 0.030429866 |
| P4HA3 | ENSG00000149380 | -3.440739883 | 0.020493809 |
| MS4A3 | ENSG00000149516 | -1.608881633 | 0.046388974 |
| CNKSR2 | ENSG00000149970 | -1.849688537 | 0.02448569 |
| LPHN3 | ENSG00000150471 | -3.166017773 | 0.00755165 |
| CACNA1C | ENSG00000151067 | -3.281501739 | 0.001337867 |
| NPAS3 | ENSG00000151322 | -4.272508906 | 0.01292435 |
| FRMD4A | ENSG00000151474 | -1.447922545 | 0.031631311 |
| ADAM8 | ENSG00000151651 | -1.344707395 | 0.001988036 |
| GABRA2 | ENSG00000151834 | -4.009691554 | 0.011473846 |
| GLT1D1 | ENSG00000151948 | -2.063585376 | 0.011565247 |
| HOMER1 | ENSG00000152413 | -2.182173995 | 0.001704655 |
| GRIA4 | ENSG00000152578 | -4.46904602 | 0.039783162 |
| PRDM8 | ENSG00000152784 | -1.378297777 | 0.006689689 |
| ZIC1 | ENSG00000152977 | -4.46904587 | 0.039780559 |
| PTPRD | ENSG00000153707 | -2.309604047 | 0.035845996 |
| HS3ST3A1 | ENSG00000153976 | -8.171452783 | 7.45E-05 |
| FAM167A | ENSG00000154319 | -2.054871353 | 0.029635718 |
| FGD5 | ENSG00000154783 | -2.597756802 | 0.047059094 |
| TMEM55A | ENSG00000155099 | -1.59404924 | 0.001048904 |
| GPR78 | ENSG00000155269 | -4.070057966 | 0.02694197 |
| VSIG4 | ENSG00000155659 | -3.297950941 | 5.46E-05 |
| ADCY8 | ENSG00000155897 | -4.373133504 | 0.043734743 |
| AFF2 | ENSG00000155966 | -1.474584098 | 0.005190484 |
| FUT6 | ENSG00000156413 | -3.109944681 | 0.047818546 |
| TDRD9 | ENSG00000156414 | -1.850393097 | 0.038070422 |
| NRG1 | ENSG00000157168 | -3.092446979 | 0.043796507 |
| KCNJ15 | ENSG00000157551 | -2.033768828 | 0.020984098 |
| CREB3L1 | ENSG00000157613 | -3.577850421 | 0.015350932 |
| SLC37A3 | ENSG00000157800 | -1.468732716 | 0.031954919 |
| DPYSL5 | ENSG00000157851 | -5.668800867 | 0.004963421 |
| DRC1 | ENSG00000157856 | -4.282931828 | 0.013605287 |
| COLEC12 | ENSG00000158270 | -3.091374495 | 0.020145389 |
| CD1B | ENSG00000158485 | -3.860452187 | 0.040225358 |
| NCF1 | ENSG00000158517 | -1.561622174 | 0.011619245 |
| NBL1 | ENSG00000158747 | -2.965147351 | 0.033826255 |
| HTR6 | ENSG00000158748 | -4.484986904 | 0.01074599 |
| CDA | ENSG00000158825 | -2.290790367 | 0.010030877 |
| C1orf51 | ENSG00000159208 | -3.724051192 | 0.010541609 |
| PTMS | ENSG00000159335 | -1.227624887 | 0.010523169 |
| PADI4 | ENSG00000159339 | -1.525972427 | 0.019434704 |
| IRX6 | ENSG00000159387 | -7.058259885 | 0.000658185 |
| CELF3 | ENSG00000159409 | -3.112067495 | 0.012715125 |
| RGL4 | ENSG00000159496 | -1.868453142 | 0.002921407 |
| ACE | ENSG00000159640 | -1.667496269 | 0.041949472 |
| TPPP3 | ENSG00000159713 | -1.735282722 | 0.042877254 |
| TFF3 | ENSG00000160180 | -3.06322037 | 0.026508461 |
| TFF2 | ENSG00000160181 | -6.131774378 | 0.007879233 |
| TFF1 | ENSG00000160182 | -6.917170394 | 0.003410477 |
| PDXK | ENSG00000160209 | -1.019102009 | 0.02406746 |
| DEDD2 | ENSG00000160570 | -1.103826534 | 0.014983769 |
| PTH1R | ENSG00000160801 | -3.722704889 | 0.027195567 |
| CELF5 | ENSG00000161082 | -2.454204634 | 0.049530682 |
| CD300LG | ENSG00000161649 | -3.383368072 | 0.024617684 |
| NAGS | ENSG00000161653 | -3.483743097 | 0.007531636 |
| ALOX15 | ENSG00000161905 | -2.496101005 | 0.049580691 |
| CCDC64B | ENSG00000162069 | -2.758838963 | 0.021193564 |
| PDZK1IP1 | ENSG00000162366 | -2.578801981 | 0.023578123 |
| PRKAA2 | ENSG00000162409 | -4.375722665 | 0.017135437 |
| FCGR3B | ENSG00000162747 | -1.948625293 | 0.011963399 |
| IER5 | ENSG00000162783 | -1.08782761 | 0.03014505 |
| ACTG2 | ENSG00000163017 | -4.438708348 | 0.003773214 |
| SGPP2 | ENSG00000163082 | -2.737463424 | 0.032283152 |
| MSX1 | ENSG00000163132 | -4.054056669 | 0.015928934 |
| S100A9 | ENSG00000163220 | -2.504836164 | 0.000215454 |
| S100A12 | ENSG00000163221 | -3.493725225 | 2.92E-06 |
| NPPC | ENSG00000163273 | -4.43772166 | 0.005704532 |
| ALPPL2 | ENSG00000163286 | -4.255950896 | 0.020545898 |
| CCKAR | ENSG00000163394 | -4.742068878 | 0.031307859 |
| MNDA | ENSG00000163563 | -1.454331627 | 0.029077977 |
| GYG1 | ENSG00000163754 | -1.061831982 | 0.005287901 |
| SLC51A | ENSG00000163959 | -2.544632281 | 0.033054984 |
| S100P | ENSG00000163993 | -2.3588472 | 0.014214124 |
| CLDN19 | ENSG00000164007 | -3.709411014 | 0.028451537 |
| CAMP | ENSG00000164047 | -2.964773766 | 0.001696359 |
| MST1R | ENSG00000164078 | -3.301818874 | 0.008037344 |
| NDST3 | ENSG00000164100 | -3.104703959 | 0.012821555 |
| HHIP | ENSG00000164161 | -2.479832846 | 0.034055819 |
| ANKRD33B | ENSG00000164236 | -1.597400304 | 0.03375214 |
| SCGB3A2 | ENSG00000164265 | -4.354858743 | 0.02560674 |
| PI16 | ENSG00000164530 | -5.17214052 | 0.005748816 |
| SYTL3 | ENSG00000164674 | -1.206078158 | 0.02975443 |
| SHH | ENSG00000164690 | -5.38181674 | 0.000241494 |
| DEFA4 | ENSG00000164821 | -4.086437387 | 1.27E-05 |
| RASEF | ENSG00000165105 | -4.293824352 | 0.016176477 |
| TRPV6 | ENSG00000165125 | -1.914823178 | 0.027942224 |
| MID1IP1 | ENSG00000165175 | -1.10191083 | 0.006784602 |
| LRRC18 | ENSG00000165383 | -5.347585493 | 0.007081564 |
| GJB2 | ENSG00000165474 | -4.413187044 | 0.008680446 |
| BEND7 | ENSG00000165626 | -3.953719156 | 0.015944351 |
| SLC18A2 | ENSG00000165646 | -3.252683525 | 0.003210813 |
| VWA2 | ENSG00000165816 | -3.491350859 | 0.049322299 |
| ABTB2 | ENSG00000166016 | -2.27152322 | 0.00166957 |
| ADAMTS15 | ENSG00000166106 | -4.226225728 | 0.012735807 |
| DCHS1 | ENSG00000166341 | -1.780674976 | 0.010836839 |
| PLD4 | ENSG00000166428 | -1.292928138 | 0.008537163 |
| PRTG | ENSG00000166450 | -4.139937751 | 0.005725499 |
| CLEC4E | ENSG00000166523 | -2.558025151 | 0.004174489 |
| B4GALNT2 | ENSG00000167080 | -4.838275395 | 0.028097657 |
| LOXHD1 | ENSG00000167210 | -2.845042724 | 0.015591575 |
| CYP2S1 | ENSG00000167600 | -3.161303228 | 0.003559642 |
| SEMA6B | ENSG00000167680 | -2.893704769 | 0.002589112 |
| HSD11B1L | ENSG00000167733 | -1.55644853 | 0.022788286 |
| KLK11 | ENSG00000167757 | -6.534454917 | 0.00259876 |
| EVPL | ENSG00000167880 | -2.023378846 | 0.01017569 |
| MGAT5B | ENSG00000167889 | -2.906120079 | 0.028886922 |
| FAM83B | ENSG00000168143 | -4.544473608 | 0.037345813 |
| XIRP1 | ENSG00000168334 | -4.402725595 | 0.007664908 |
| TXNDC2 | ENSG00000168454 | -2.755349752 | 0.023342439 |
| RAB31 | ENSG00000168461 | -1.695135782 | 0.003306818 |
| DPCR1 | ENSG00000168631 | -7.187094302 | 0.001170368 |
| SHOX2 | ENSG00000168779 | -3.114814041 | 0.01162528 |
| HTR1E | ENSG00000168830 | -4.343478371 | 0.043161967 |
| BTNL3 | ENSG00000168903 | -4.995915314 | 0.020686793 |
| MAP2K1 | ENSG00000169032 | -1.078457809 | 0.014972085 |
| ARMC4 | ENSG00000169126 | -2.9522171 | 0.02564599 |
| MN1 | ENSG00000169184 | -1.244573735 | 0.045358524 |
| RAB3B | ENSG00000169213 | -3.053128023 | 0.018085992 |
| CCDC8 | ENSG00000169515 | -3.685358975 | 0.009105567 |
| GKN1 | ENSG00000169605 | -7.552854977 | 0.001441154 |
| CHRNA5 | ENSG00000169684 | -3.20575936 | 0.007500789 |
| ROBO1 | ENSG00000169855 | -5.088057484 | 0.000258049 |
| MUC17 | ENSG00000169876 | -5.20594631 | 0.019844122 |
| FABP4 | ENSG00000170323 | -2.791225414 | 0.038894445 |
| CST1 | ENSG00000170373 | -5.375847763 | 0.017051509 |
| LRRN2 | ENSG00000170382 | -2.58873072 | 0.031213813 |
| KRT8 | ENSG00000170421 | -1.688045303 | 0.043595677 |
| GTSF1 | ENSG00000170627 | -3.356986874 | 0.000472777 |
| GPR27 | ENSG00000170837 | -1.06944615 | 0.027273792 |
| LILRA3 | ENSG00000170866 | -3.463825167 | 0.011238513 |
| SOX7 | ENSG00000171056 | -4.433863762 | 0.002846415 |
| SOSTDC1 | ENSG00000171243 | -3.965278637 | 0.007099875 |
| KRT19 | ENSG00000171345 | -5.822699831 | 0.008380372 |
| KRT15 | ENSG00000171346 | -3.533100346 | 0.044349619 |
| MAP1LC3B2 | ENSG00000171471 | -1.293876243 | 0.033905744 |
| BCL2L1 | ENSG00000171552 | -1.061184734 | 0.030455264 |
| SPSB1 | ENSG00000171621 | -2.317826117 | 0.04263455 |
| REG3A | ENSG00000172016 | -5.205945869 | 0.019835213 |
| CLEC12A | ENSG00000172322 | -1.119777002 | 0.019818553 |
| BPGM | ENSG00000172331 | -1.836418099 | 0.005396793 |
| CSDC2 | ENSG00000172346 | -6.544013437 | 0.00172867 |
| FUT9 | ENSG00000172461 | -4.3836159 | 0.025691656 |
| HOXC5 | ENSG00000172789 | -7.139415077 | 0.003485061 |
| CCDC96 | ENSG00000173013 | -1.451938696 | 0.049186052 |
| STOX2 | ENSG00000173320 | -2.296294606 | 0.042256108 |
| OLR1 | ENSG00000173391 | -2.115056688 | 0.029578615 |
| PTPRM | ENSG00000173482 | -2.506996996 | 0.001441846 |
| TNFRSF10C | ENSG00000173535 | -2.278148438 | 0.000983524 |
| C2orf70 | ENSG00000173557 | -4.402298013 | 0.030253875 |
| GPR64 | ENSG00000173698 | -4.463216684 | 0.032520563 |
| MUC13 | ENSG00000173702 | -4.584532039 | 0.035106246 |
| PHOSPHO1 | ENSG00000173868 | -2.23222395 | 0.006944947 |
| GPR160 | ENSG00000173890 | -1.639268823 | 0.003055275 |
| SLCO4C1 | ENSG00000173930 | -1.750075805 | 0.001599641 |
| SPERT | ENSG00000174015 | -4.032241388 | 0.034703066 |
| SLC26A9 | ENSG00000174502 | -3.201367766 | 0.0329192 |
| SLCO2A1 | ENSG00000174640 | -4.226357605 | 0.009493381 |
| BTC | ENSG00000174808 | -3.379323406 | 0.035791522 |
| SEZ6L2 | ENSG00000174938 | -2.907472881 | 0.040685965 |
| P2RY14 | ENSG00000174944 | -1.813982766 | 0.043202336 |
| CD164L2 | ENSG00000174950 | -3.774891399 | 0.00750384 |
| CHST2 | ENSG00000175040 | -1.364067696 | 0.033942953 |
| NR2F1 | ENSG00000175745 | -3.594205982 | 0.005344003 |
| LRRN1 | ENSG00000175928 | -2.727172147 | 0.003462646 |
| GPX2 | ENSG00000176153 | -5.131965581 | 0.021773984 |
| B3GNT4 | ENSG00000176383 | -2.360953247 | 0.02773433 |
| CLEC14A | ENSG00000176435 | -2.019625292 | 0.034749582 |
| B3GNT5 | ENSG00000176597 | -1.132744873 | 0.005511443 |
| BOK | ENSG00000176720 | -4.302454457 | 0.007405196 |
| SCN4B | ENSG00000177098 | -3.920311481 | 0.013650658 |
| TRIM72 | ENSG00000177238 | -2.60572529 | 0.034595253 |
| CASKIN2 | ENSG00000177303 | -1.853797154 | 0.029119864 |
| C8orf47 | ENSG00000177459 | -3.44302179 | 0.031992061 |
| PLEC | ENSG00000178209 | -2.041772554 | 0.003017527 |
| SH2D4B | ENSG00000178217 | -2.483455999 | 0.026797876 |
| RPP25 | ENSG00000178718 | -1.511955995 | 0.032964882 |
| GP5 | ENSG00000178732 | -2.074868468 | 0.018871803 |
| EXOSC4 | ENSG00000178896 | -1.251551465 | 0.016568956 |
| NSUN7 | ENSG00000179299 | -3.078289983 | 0.005776657 |
| FJX1 | ENSG00000179431 | -4.560513244 | 0.012650655 |
| MKRN3 | ENSG00000179455 | -2.579334695 | 0.003931562 |
| OR9A2 | ENSG00000179468 | -3.226806941 | 0.03966377 |
| ABCA13 | ENSG00000179869 | -3.045089455 | 1.21E-06 |
| GPR144 | ENSG00000180264 | -5.584399949 | 0.014187609 |
| HOXC9 | ENSG00000180806 | -3.436271714 | 0.042147531 |
| HOXC10 | ENSG00000180818 | -4.491297097 | 0.018132654 |
| CXCR2 | ENSG00000180871 | -1.423271076 | 0.032189062 |
| CUEDC1 | ENSG00000180891 | -1.620929388 | 0.019679525 |
| CCDC108 | ENSG00000181378 | -4.07659261 | 0.006043872 |
| AATK | ENSG00000181409 | -1.70100082 | 0.031808134 |
| ZNF467 | ENSG00000181444 | -1.277634195 | 0.006026549 |
| TMEM45A | ENSG00000181458 | -2.788763776 | 0.004916848 |
| P2RY13 | ENSG00000181631 | -1.318262641 | 0.024907322 |
| TNFSF15 | ENSG00000181634 | -3.09810187 | 0.008342611 |
| PHLDA2 | ENSG00000181649 | -2.212901585 | 0.046175661 |
| RGMA | ENSG00000182175 | -3.187717871 | 0.038953339 |
| LDOC1 | ENSG00000182195 | -3.634483447 | 0.009711206 |
| B4GALNT4 | ENSG00000182272 | -3.737254985 | 0.015662828 |
| LIPF | ENSG00000182333 | -8.468463512 | 0.001010922 |
| FAM27A | ENSG00000182368 | -3.092553441 | 0.014141176 |
| MXRA7 | ENSG00000182534 | -1.871559578 | 0.000694941 |
| NXPH3 | ENSG00000182575 | -3.190982337 | 0.02979289 |
| NDN | ENSG00000182636 | -2.08853494 | 0.030259573 |
| PAPPA | ENSG00000182752 | -2.158963935 | 0.047036254 |
| MAFA | ENSG00000182759 | -2.230200964 | 0.039314661 |
| COL18A1 | ENSG00000182871 | -1.512725933 | 0.018682275 |
| GPR97 | ENSG00000182885 | -1.584557859 | 0.029705871 |
| GJC1 | ENSG00000182963 | -3.033922932 | 0.020506604 |
| C19orf59 | ENSG00000183019 | -1.804091843 | 0.019094909 |
| SLC25A21 | ENSG00000183032 | -3.272323047 | 0.018467123 |
| CALN1 | ENSG00000183166 | -2.703859781 | 0.026210818 |
| C2CD4C | ENSG00000183186 | -3.613572227 | 0.04620003 |
| RIPK4 | ENSG00000183421 | -5.204480766 | 0.009030211 |
| PGPEP1L | ENSG00000183571 | -4.062927775 | 0.036015706 |
| TNFAIP8L3 | ENSG00000183578 | -3.938839247 | 0.033902573 |
| GKN2 | ENSG00000183607 | -5.76655837 | 0.01173049 |
| MRC1L1 | ENSG00000183748 | -2.523210694 | 0.025067601 |
| B3GALT5 | ENSG00000183778 | -5.289956626 | 0.010642832 |
| FAM3B | ENSG00000183844 | -4.29213593 | 0.014897387 |
| SDR42E2 | ENSG00000183921 | -1.410794717 | 0.032288318 |
| TMPRSS2 | ENSG00000184012 | -7.343144286 | 0.002167735 |
| CLDN5 | ENSG00000184113 | -3.289314222 | 0.023661088 |
| TACSTD2 | ENSG00000184292 | -5.626527089 | 2.27E-05 |
| PRKD1 | ENSG00000184304 | -4.291084566 | 0.002198364 |
| SLIT3 | ENSG00000184347 | -4.008630531 | 0.012265458 |
| FOXD4L1 | ENSG00000184492 | -2.158138109 | 0.022079101 |
| CEND1 | ENSG00000184524 | -4.122348075 | 0.040028382 |
| 5-Sep | ENSG00000184702 | -1.071015168 | 0.028070659 |
| OSBP2 | ENSG00000184792 | -1.701424419 | 0.026668914 |
| MUC6 | ENSG00000184956 | -3.670679928 | 0.016594907 |
| HIST1H2BL | ENSG00000185130 | -1.020222578 | 0.002466859 |
| FAM47A | ENSG00000185448 | -4.58453107 | 0.035089024 |
| PRKG1 | ENSG00000185532 | -2.656210402 | 0.038379463 |
| NR2F2 | ENSG00000185551 | -2.855833856 | 0.017309694 |
| AHNAK2 | ENSG00000185567 | -3.336896011 | 0.012214492 |
| OLFML2A | ENSG00000185585 | -1.868382046 | 0.03795947 |
| PRAME | ENSG00000185686 | -6.494249425 | 1.57E-05 |
| SRL | ENSG00000185739 | -3.305547651 | 0.046087777 |
| SLC52A2 | ENSG00000185803 | -1.012454995 | 0.019445515 |
| ATP4B | ENSG00000186009 | -7.917101532 | 0.001161577 |
| DLEU7 | ENSG00000186047 | -2.17758145 | 0.031076544 |
| CYP4X1 | ENSG00000186377 | -4.164771788 | 0.017706849 |
| FCAR | ENSG00000186431 | -1.614757524 | 0.045700566 |
| SPATA12 | ENSG00000186451 | -3.582306421 | 0.048731211 |
| CYP4F3 | ENSG00000186529 | -1.898794341 | 0.003445521 |
| PRG2 | ENSG00000186652 | -2.265467499 | 0.019735504 |
| MAPT | ENSG00000186868 | -2.601995582 | 0.045373922 |
| TNFRSF18 | ENSG00000186891 | -3.770312494 | 0.004746207 |
| KANK3 | ENSG00000186994 | -4.15567465 | 0.007721061 |
| FAM9C | ENSG00000187268 | -3.601402833 | 0.032795379 |
| PCDHB13 | ENSG00000187372 | -2.271750227 | 0.025454746 |
| COL4A1 | ENSG00000187498 | -2.25607287 | 0.031823147 |
| C1orf170 | ENSG00000187642 | -4.241191882 | 0.035435958 |
| RP11-723O4.6 | ENSG00000187695 | -2.958341484 | 0.018701309 |
| KBTBD12 | ENSG00000187715 | -5.279383687 | 0.006589943 |
| DNAJB13 | ENSG00000187726 | -3.731866135 | 0.046189782 |
| PALM3 | ENSG00000187867 | -3.599357362 | 0.042890847 |
| RILPL1 | ENSG00000188026 | -1.810446285 | 0.036414122 |
| HES4 | ENSG00000188290 | -3.661288047 | 0.024702499 |
| HBA2 | ENSG00000188536 | -2.370805226 | 0.006353165 |
| NKAIN2 | ENSG00000188580 | -4.851794472 | 0.004302256 |
| S100A16 | ENSG00000188643 | -4.607445184 | 0.002966425 |
| FAM221A | ENSG00000188732 | -1.378537663 | 0.03182295 |
| BCL2L15 | ENSG00000188761 | -1.959708087 | 0.01321585 |
| BEND4 | ENSG00000188848 | -2.713065248 | 0.010358673 |
| FAM196A | ENSG00000188916 | -4.538088499 | 0.037719172 |
| VSTM1 | ENSG00000189068 | -1.49354368 | 0.044233664 |
| ANKRD34B | ENSG00000189127 | -2.963680009 | 0.044555053 |
| PLAC9 | ENSG00000189129 | -4.572143877 | 0.024972211 |
| SYCP2 | ENSG00000196074 | -2.079293565 | 0.035294543 |
| SPOCK3 | ENSG00000196104 | -3.656689079 | 0.024452901 |
| ZNF676 | ENSG00000196109 | -4.656503383 | 0.026408748 |
| MYT1 | ENSG00000196132 | -2.612959577 | 0.013530095 |
| SERPINA3 | ENSG00000196136 | -5.354887962 | 0.005976054 |
| FAT4 | ENSG00000196159 | -1.971392885 | 0.032413321 |
| NUDT11 | ENSG00000196368 | -3.437778852 | 0.044927715 |
| HIST1H2BM | ENSG00000196374 | -1.270889783 | 0.00569174 |
| PRTN3 | ENSG00000196415 | -2.0988288 | 0.041004371 |
| FGF16 | ENSG00000196468 | -3.666082532 | 0.017068822 |
| MME | ENSG00000196549 | -1.780647747 | 0.00989514 |
| HBG2 | ENSG00000196565 | -2.652918233 | 0.021180353 |
| AJAP1 | ENSG00000196581 | -3.147588679 | 0.030761303 |
| UGT2B15 | ENSG00000196620 | -4.979319323 | 0.014286943 |
| ZNF239 | ENSG00000196793 | -1.566495294 | 0.035116018 |
| PCDHB16 | ENSG00000196963 | -3.763547095 | 0.009356934 |
| HIST1H4J | ENSG00000197238 | -2.185370343 | 0.002649175 |
| OR6N1 | ENSG00000197403 | -4.00772575 | 0.033186244 |
| CYP2F1 | ENSG00000197446 | -3.507521705 | 0.043771465 |
| MFAP5 | ENSG00000197614 | -3.849422024 | 0.043829639 |
| C1orf122 | ENSG00000197982 | -1.403032884 | 0.027840233 |
| AKR1B10 | ENSG00000198074 | -5.584399443 | 0.014176703 |
| SULT1C2 | ENSG00000198203 | -4.259185555 | 0.006503925 |
| HOXC4 | ENSG00000198353 | -5.305957516 | 0.000619019 |
| PIM3 | ENSG00000198355 | -1.694605612 | 0.004629954 |
| ZNF536 | ENSG00000198597 | -7.930552678 | 0.000777916 |
| PPP1R14C | ENSG00000198729 | -4.063703236 | 0.030887284 |
| MSRB1 | ENSG00000198736 | -1.163289488 | 0.013939483 |
| DCAF12 | ENSG00000198876 | -1.171855477 | 0.021538892 |
| ASB12 | ENSG00000198881 | -3.66982444 | 0.024736365 |
| CAPN8 | ENSG00000203697 | -5.862731752 | 0.010008317 |
| HIST2H2AA4 | ENSG00000203812 | -2.236875439 | 0.001515962 |
| HIST2H3A | ENSG00000203852 | -2.984264389 | 0.000709519 |
| NEU4 | ENSG00000204099 | -1.6203907 | 0.032485252 |
| NAP1L6 | ENSG00000204118 | -3.721808899 | 0.021607568 |
| C10orf128 | ENSG00000204161 | -1.400400014 | 0.038789005 |
| BMPR2 | ENSG00000204217 | -1.02920602 | 0.040074995 |
| HSPA1A | ENSG00000204389 | -1.301095525 | 0.019615154 |
| POU5F1 | ENSG00000204531 | -4.956628396 | 0.005276671 |
| PSORS1C1 | ENSG00000204540 | -4.39683622 | 0.006648139 |
| MROH6 | ENSG00000204839 | -1.116133841 | 0.038600823 |
| SYCE1L | ENSG00000205078 | -2.387334664 | 0.035361727 |
| C11orf91 | ENSG00000205177 | -3.234420154 | 0.029129567 |
| VIT | ENSG00000205221 | -7.27277333 | 0.000242685 |
| GOLGA6L10 | ENSG00000205281 | -2.725271805 | 0.032663911 |
| DEFA1 | ENSG00000206047 | -4.339126986 | 0.000240569 |
| HBA1 | ENSG00000206172 | -1.959466894 | 0.013016979 |
| HBM | ENSG00000206177 | -2.806844166 | 0.009200689 |
| LTB4R2 | ENSG00000213906 | -2.44112397 | 0.020799139 |
| MUC5AC | ENSG00000215182 | -6.941638043 | 0.003527228 |
| FAM19A5 | ENSG00000219438 | -5.872015041 | 0.000859726 |
| HEPN1 | ENSG00000221932 | -4.092306834 | 0.030578281 |
| CTAGE4 | ENSG00000225932 | -3.223958703 | 0.019156895 |
| ORM2 | ENSG00000228278 | -3.939313907 | 0.020790403 |
| PGA4 | ENSG00000229183 | -7.098298897 | 0.003870431 |
| ORM1 | ENSG00000229314 | -3.17167077 | 0.005712516 |
| PGA3 | ENSG00000229859 | -7.386177124 | 0.002471678 |
| TNFSF12 | ENSG00000239697 | -1.515542377 | 0.025797114 |
| DEFA3 | ENSG00000239839 | -3.549810601 | 0.001462375 |
| C1orf226 | ENSG00000239887 | -2.1565952 | 0.037863355 |
| CT45A5 | ENSG00000242284 | -6.982756554 | 0.002562887 |
| RP11-508N12.4 | ENSG00000242631 | -4.225065449 | 0.006316401 |
| PPAN-P2RY11 | ENSG00000243207 | -5.808745027 | 0.000663596 |
| RGAG1 | ENSG00000243978 | -3.867099372 | 0.021199204 |
| CFB | ENSG00000244255 | -3.734513755 | 0.040838492 |
| HBB | ENSG00000244734 | -1.729702176 | 0.026100646 |
| RP11-382J12.1 | ENSG00000246366 | -2.766510316 | 0.028380185 |
| MTRNR2L5 | ENSG00000249860 | -4.205935104 | 0.047416275 |
| IQCJ-SCHIP1 | ENSG00000250588 | -2.700346735 | 0.005833685 |
| SHANK3 | ENSG00000251322 | -1.451280966 | 0.029012163 |
| TRNP1 | ENSG00000253368 | -5.595536623 | 0.000198357 |
| AP000783.1 | ENSG00000254667 | -2.852319251 | 0.024709538 |
| NOX5 | ENSG00000255346 | -3.686203438 | 0.023177031 |
| AGAP2-AS1 | ENSG00000255737 | -2.984514839 | 0.004320635 |
| RP13-512J5.1 | ENSG00000255767 | -3.551525608 | 0.025375657 |
| CCDC177 | ENSG00000255994 | -4.584530172 | 0.035073079 |
| PGA5 | ENSG00000256713 | -8.75044949 | 0.000185244 |
| HP | ENSG00000257017 | -2.604672678 | 0.000351298 |
| MGAM | ENSG00000257335 | -2.164471562 | 2.16E-05 |
| OVCH1-AS1 | ENSG00000257599 | -3.980810835 | 0.042343797 |
| CLEC5A | ENSG00000258227 | -2.416296481 | 0.000809786 |
| SPESP1 | ENSG00000258484 | -4.255800728 | 0.033363008 |
| RP11-463D19.2 | ENSG00000258677 | -2.657391015 | 0.020398054 |
| GALT | ENSG00000258728 | -4.812210163 | 0.030133815 |
| MC1R | ENSG00000258839 | -1.045240363 | 0.049981723 |
| PCDHGA4 | ENSG00000262576 | -2.826257086 | 0.029734319 |
| CHTF8 | ENSG00000263203 | -4.232223083 | 0.016775146 |
| CTD-2132N18.3 | ENSG00000267261 | -4.381489971 | 0.036170273 |
| CTD-2528L19.4 | ENSG00000267552 | -4.071840213 | 0.022529718 |
| CTD-2105E13.6 | ENSG00000267706 | -4.724480778 | 0.003767739 |
| CTD-2207O23.12 | ENSG00000267952 | -7.511795041 | 0.000387077 |
| AC132192.1 | ENSG00000268403 | -3.690917778 | 0.049213207 |
| DNAH17-AS1 | ENSG00000268470 | -4.09749578 | 0.021133433 |
| AL133373.1 | ENSG00000268657 | -5.129535834 | 0.00712337 |
| AC093323.1 | ENSG00000268791 | -4.518191822 | 0.013003042 |
| FBXO17 | ENSG00000269190 | -5.082530994 | 0.011143339 |
| CTD-2192J16.22 | ENSG00000269242 | -3.932303231 | 0.023734158 |
| CTD-3148I10.9 | ENSG00000269469 | -3.960981454 | 0.018593204 |
| AC096677.1 | ENSG00000269690 | -3.765485079 | 0.006171821 |
| RP11-248J23.6 | ENSG00000269948 | -2.432030732 | 0.035868514 |
| RP5-850E9.3 | ENSG00000270299 | -5.087586374 | 0.01883331 |
| DOC2B | ENSG00000272636 | -4.522733958 | 0.002555086 |
| RP11-834C11.12 | ENSG00000273049 | -4.386741827 | 0.048316395 |

Table S5 Pathway analyses of clinical AML samples associated with CPT1A expression

| Pathways in cancer | Raw p |
| --- | --- |
| Serotonergic synapse | 1.60E-06 |
| Staphylococcus aureus infection | 4.94E-06 |
| Axon guidance | 4.41E-05 |
| Protein digestion and absorption | 0.000202 |
| MAPK signaling pathway | 0.000462 |
| Estrogen signaling pathway | 0.000975 |
| cGMP-PKG signaling pathway | 0.00146 |
| Neuroactive ligand-receptor interaction | 0.001717 |
| IL-17 signaling pathway | 0.002211 |
| Parathyroid hormone synthesis, secretion and action | 0.002271 |
| Signaling pathways regulating pluripotency of stem cells | 0.00244 |
| Oxytocin signaling pathway | 0.002598 |
| Bladder cancer | 0.003196 |
| Vascular smooth muscle contraction | 0.003648 |
| Human T-cell leukemia virus 1 infection | 0.003721 |
| African trypanosomiasis | 0.004811 |
| Prostate cancer | 0.005207 |
| Pathways in cancer | 0.00626 |
| Gastric acid secretion | 0.006356 |
| Retrograde endocannabinoid signaling | 0.008434 |
| Malaria | 0.010412 |
| Insulin secretion | 0.010595 |
| Glutamatergic synapse | 0.010622 |
| Amphetamine addiction | 0.012463 |
| Proteoglycans in cancer | 0.015604 |
| Circadian entrainment | 0.015828 |
| PI3K-Akt signaling pathway | 0.016066 |
| cAMP signaling pathway | 0.01648 |
| EGFR tyrosine kinase inhibitor resistance | 0.016552 |
| Salivary secretion | 0.016591 |
| Endocrine resistance | 0.01738 |
| Rap1 signaling pathway | 0.017702 |
| AGE-RAGE signaling pathway in diabetic complications | 0.018195 |
| Relaxin signaling pathway | 0.018631 |
| ErbB signaling pathway | 0.022578 |
| Cushing syndrome | 0.02344 |
| Dopaminergic synapse | 0.025311 |
| Glycosaminoglycan biosynthesis - heparan sulfate / heparin | 0.02541 |
| Long-term depression | 0.027524 |
| Nicotine addiction | 0.028207 |
| TGF-beta signaling pathway | 0.028609 |
| Fluid shear stress and atherosclerosis | 0.029212 |
| Small cell lung cancer | 0.02993 |
| Cholinergic synapse | 0.033815 |
| Melanoma | 0.034013 |
| Phagosome | 0.035812 |
| Transcriptional misregulation in cancer | 0.041081 |
| Adrenergic signaling in cardiomyocytes | 0.043353 |
| Osteoclast differentiation | 0.043475 |
| Apoptosis - multiple species | 0.043541 |
| NF-kappa B signaling pathway | 0.045371 |
| Glycosphingolipid biosynthesis - lacto and neolacto series | 0.049637 |
